# Nanoparticle delivery of TFOs is a novel targeted therapy for HER2 amplified breast cancer

**DOI:** 10.1186/s12885-023-11176-8

**Published:** 2023-07-20

**Authors:** Xiaojing Yang, Yi Xu, Jie Fu, Zan Shen

**Affiliations:** 1 Department of Oncology, Shanghai Sixth People’s Hospital Affiliated to Shanghai Jiao Tong University School of Medicine, No. 600, Yishan Road, Shanghai, 200233 China; 2 Department of Radiation Oncology, Shanghai Sixth People’s Hospital Affiliated to Shanghai Jiao Tong University School of Medicine, Shanghai, 200333 China

**Keywords:** *HER2*, TFO, Polyamino acid nanomaterials, Breast cancer, Apoptosis

## Abstract

**Purpose:**

The human *EGFR2* (*HER2*) signaling pathway is one of the most actively studied targets in cancer transformation research. Ttriplex-forming oligonucleotides (TFOs) activate DNA damage and induce apoptosis. We aim to encapsulate TFO-HER2 with nano-particle ZW-128 to suppress breast cell growth in vitro and in vivo.

**Experimental design:**

We designed a set of TFO fragments targeting HER2 and verified their effectiveness. We encapsulated TFO-HER2 in ZW-128 to form nano-drug TFO@ZW-128. Cell counting kit 8, flow cytometry, and western blotting were used to evaluate the effect of TFO@ZW-128 on cell proliferation and the expressions of related proteins. The ant-itumor effect of TFO@ZW-128 was evaluated in vivo using nude mice breast cancer model.

**Results:**

TFO@ZW-128 had efficient cellular uptake in amplified HER2 breast cancer cells. TFO@ZW-128 showed an 80-fold increase in TFO utilization compared with TFO-HER2 in the nude mouse breast cancer model. Meanwhile, TFO@ZW-128 dramatically inhibited the growth of HER2-overexpressing tumors compared with TFO-HER2 (*P* < 0.05). Furthermore, TFO@ZW-128-induced cell apoptosis was in a p53-independent manner.

**Conclusions:**

In this study, we designed nano-drug TFO@ZW-128, which has proven effective and non-toxic in targeted therapy for ectopic HER2-expressing tumors.

**Supplementary Information:**

The online version contains supplementary material available at 10.1186/s12885-023-11176-8.

## Introduction

HER2-positive patients account for 15% -20% of the total breast cancer population, with aggressive, high recurrence rates and low survival rates [1]. The activation of HER2 function can promote tumorigenesis [2]. In recent years, various anti-HER2 targeted therapies have brought new challenges and hopes to HER2-positive patients [3]. However, patients with HER2-positive breast cancer have been reported to develop relapse and resistance even after treatment with existing targeted HER2 agents [4]. Therefore, new clinical treatment strategies are urgently needed to improve patient survival further.

Triplex-forming oligonucleotide (TFO) is a therapeutic strategy for target gene amplification [5, 6]. The binding of TFO within the double-helix central groove causes DNA perturbation, hinders replication fork progression, and leads to DNA double-strand break (DSB) [7]. Thus, the formation of multiple chromosomal triple-stranded structures can induce sufficient DNA damage to activate apoptosis [8]. The Nucleotide excision repair (NER) pathway resolves low levels of triplex-induced DNA damage so that normal cells can tolerate TFO treatment [9]. Tiwari et al. developed a TFO that binds to the *HER2* gene in a sequence-specific manner [10]. Their studies found that TFO promoted apoptosis in HER2-overexpressing breast and ovarian cancer cells in vitro and in vivo but had no effect on *HER2* gene expression. However, there are still some challenges in delivering TFO to target cells. Tiwari et al. and Jiao et al. used nano-particles to transfer TFO fragments [10, 11]. The main obstacles of nanoparticles are enzyme degradation, short half-life, and poor cellular uptake [12]. Therefore, it is necessary to find a better gene delivery system. Compared with other biomedical polymer materials, Poly-amino-acids have the same main chain structure (peptide bond) as natural proteins and can form stable secondary structures (such as α-helix, β-fold, etc.) [13], which are ideal synthetic polymers for simulating protein structure and function due to its rich selection of amino acids [14]. Poly-amino-acid materials have excellent biocompatibility, biodegradability, and biological activity and have demonstrated good application prospects in various fields, including antibacterial, antifouling, anti-tumor, gene delivery, tissue engineering, and immunomodulation [15, 16]. Therefore, the application of amino acid-based nanomaterials in TFO drug delivery systems is of great significance for the clinical application of gene-targeted drug delivery systems.

In this study, the branched polyurethane polymer ZW-128 was donated from the Institute of Ultrasound in our hospital by Dr. Jianrong Wu. TFO@ZW-128 was formed using the ZW-128 package containing the TFO target fragment of our design. We aimed to explore the anti-tumor effects of the polymeric nanomedicine TFO@ZW-128 and further validate the anti-tumor effects of this delivery system in high-expressing HER2 tumors in vivo.

## Material and methods

### Design of HER2-TFO

HER2-11074, a 26-NT chromosome 17, is located between exon 23 and exon 24 of the *HER2* gene at the positions of 39725858-39725883. The specific sequences were: 5'-GGTCCGTCGGTCGTGTCGAGTCGGTG-3'. Paired free energy sizes were calculated with Snapgene, DNAman, and NovoPro comparisons according to the Watson–Crick pairing, with the intermediate base adjusted according to the Hoogsteen pairing. The control mixed-sequence oligonucleotide Mix26: AAGGTTCCAGTCAAGGTTCCACTGAA.

### Branched polyamino acid polymer ZW-128

Branched polyamino acid polymer ZW-128 was a gift from Dr. Wu Jianrong from the Ultrasound Institute of Shanghai Sixth People's Hospital. Due to the pending patent, the precise composition and preparation technique cannot be shown. The ZW-128 is about 120 to 145 nm in size and has an average potential of + 12.5 mv. Its stability is about 8 months and 4 °C preservation.

### Cells culture and transfection

BT474, SKBR3, MDA-MB-453, MCF-7, and BT20 cells (purchased from the Shanghai Chinese Academy of Sciences) were cultured routinely. BT474 cells (5 × 10^5^ cells per 2 ml) were grown in RPMI1640, along with 10% fetal bovine serum (FBS) in Nunclon 6-well plates. SKBR3 cells were grown in McCoy's 5A medium (5 × 10^5^ cells per2 ml) (Procell) containing 10% FBS in Nunclon 6-well culture plates. MDA-MB-453, MCF-7, and BT20 cells were cultured in a medium of DMEM (5 × 10^5^ cells per 2 ml) containing 10% FBS in Nunclon 6-well plates. When the fusion degree of cells reached 70% ~ 80%, the cells were transfected. The dosage of transfection reagent and TFO was adjusted according to the requirements of each group. The transfection method was performed according to Lip2000 reagent instructions.

### Flow cytometry to determine transfection efficiency

The flow cytometry was set at 557 nm as the excitation wavelength and 583 nm as the detection wavelength in PE channel. 30,000 cells were detected in each tube. Flow cytometry was used to determine the proportion of cells with TAMRA (cells transfected with TFO) due to the yellow fluorescent protein (TAMRA) in TFO. The labeled cells were collected and analyzed using CytoFLEX S (Beckman Coulter) and CytExpert software, respectively.

### TFO@ZW-128 characterization assay

The size, distribution and zeta potential of nanoparticles were measured using dynamic laser light scattering (DLS) (Malvern Zetasizer Nano ZS90) at 25 °C. 1 ~ 2 drops of nano-particle dispersion were dropped on the copper mesh with support film-coated. After the appropriate amount of methanol of TFO@ZW-128 was dissolved and dispersed evenly by ultrasound, 2μL solution was dropped on the corresponding silicon wafer and copper net, and the micromorphology was scanned and observed by transmission electron microscopy (TEM) (FEI Tecnai F20).

### Anti-tumor effect in vitro

Cell proliferation activity was measured by CCK8. The anti-proliferation effect was evaluated by cell counting kit 8(CCK-8, DOJINDO, Japan). Cells were seeded in 96-well plates at a density of 1 × 10^4^ cells per well and incubated at 37 °C for 24 h. The cells were then treated with TFO@ZW-128 for 72 h. After adding 10 µl of CCK-8 into each well and culturing for 2 h, the anti-proliferation effect of different drugs and concentrations showed a curve. Apoptosis was detected by flow cytometry. The processed cells were treated with 30 ul Binding Buffer (Bender Medsystus) and 5 ul PI (Sigma) at room temperature for 15 min without light. Saline was added to 1 ml, and Flow cytometry was applied. To detect clonogenic cell ability, HeLa cells at the logarithmic growth stage were inoculated into 6 cm-diameter dishes with 100 cells per dish. TFO@ZW-128-treated cells were given after 24 h. The clonal culture period lasted one week. The cells were fixed with 100% methanol at 4 °C for 20 min and stained with 0.2% crystal violet for 30 min at room temperature. Cells were cleaned with PBS and dried at room temperature before photographs were taken.

### Live/dead assay

The anti-tumor effects of different therapeutic schemes were demonstrated by the live/dead test. The cells were stained with calcein-AM (5 μg mL^−1^) and PI (10 μg mL^−1^) for 20 min. Cells were rinsed with PBS, and then a Fluorescence microscope was used for observation.

### Flow cytometry

The processed cells were collected and then incubated with anti-pATM (S1981; Abcam, cat#: ab81292) for 1 h at room temperature in a dilution of 1:100 in PBST. Cells were rinsed with PBST and incubated with anti-rabbit IgG H&L (Alexa Fluor® 488; Abcam, cat#: ab150077) at 1:100 dilution for 1 h at room temperature, followed by rinsing with PBST. The labeled cells were collected and analyzed using Flow cytometry and CytExpert software.

### Western blot analysis

Cells were collected and lysed 12 h after transfection. Proteins were detected by a standard protocol using the following primary antibodies: cleaved PARP (Cell Signaling, cat#: 5625), cleaved caspase-3 (Abcam, cat#: ab32042), pH2AX (S139; Abcam, cat#: ab81299), pChk1 (S345; Abcam, cat#: ab58567), Chk1 (Abcam, cat#: ab40866), pChk2 (T68; BOSTER, cat#: P00277), Chk2 (Abcam, cat#: ab109413), p53(Abcam, cat#: ab26), pH2AX (Y142; Bio-Rad, cat#: AHP3045), pHER2 (Y11221/1222; Abcam, cat#: ab131102), HER2 (Abcam, cat#: ab134182), and β-Actin (Abcam, cat#: ab115777). Horseradish peroxidase (HRP)-labeled sheep anti-rabbit secondary antibody (Jackson, cat#:111–035-003) was incubated at room temperature for 2 h. The film was placed in a fully automated chemiluminescence instrument (Tanon 5200) for imaging, and the optimal image was saved for analysis (analysis system: TanonImage).

### Animal and tumor models

All animal experiments were approved by the Shanghai Sixth People's Hospital Animal Ethics Committee. All the 5-week-old female BALB/C nude mice used in the experiment were purchased from Slake Experimental Animal Co., Ltd. (Shanghai). To establish the BT474 nude mice tumor model, BT474 cells (2.5 × 10^7^) were injected subcutaneously into the right hind limb of nude mice. When the tumor volume increased to about 100mm^3^, tumor-bearing nude mice were randomly divided into four groups, including (1) PBS, (2) TFO, (3) ZW-128, and (4) TFO@ZW-128. The different preparations (100 μL) were injected into the tumor on days 0, 2, 4, 6, 8, 10, 12. Tumor size and weight were recorded every other day after treatments.

### Efficacy and toxicity in vivo

After 14 days of treatment and 24 days of observation, all tumor specimens were collected from different groups of nude mice. After fixation in 4% paraformaldehyde, tumor tissue was sectioned to 4 μm thickness for hematoxylin and eosin (H & E). The heart, liver, spleen, lung, and kidney were stained with H & E.

### Statistical analysis

The statistical analysis was performed using SPSS 21.0 (IBM, Armonk, NY, USA). The significance of differences was determined using the variance analysis (ANOVA) and Tukey's post hoc test as indicated. Continuous data were expressed as mean ± standard deviation (SD). Analysis of variance showed that *P < 0.05 was statistically significant.

## Results

### Verify the role of TFO in breast cancer cells

#### Fluorescence confocal detection of TFO into the nucleus

**Fig. 1:**
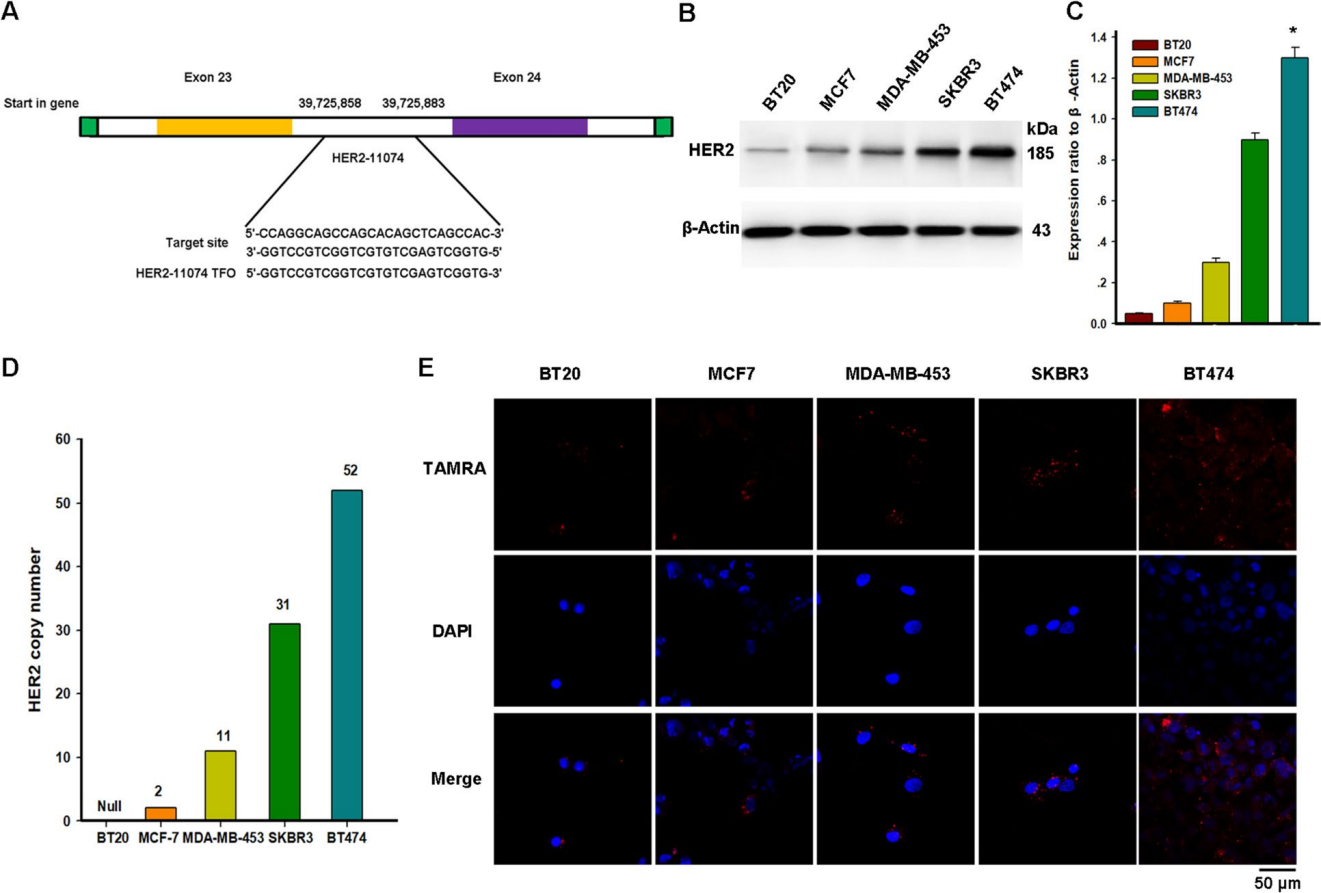
Uptake of TFO by different breast cancer cell lines. **A**. TFO is designed to bind to a polypurine sequence located in the exon of the *HER2* gene. The HER2-11074 used in this article was designed to bind to a region between exon 23 and 24. **B**. Western blot analysis of HER2 protein levels in breast cancer cell lines with different gene copy numbers. **C**. Quantification indicated the level of HER2 in these cells. Data are presented as mean ± SEM and were analyzed by Tukey's post hoc test; Error bars represent SEM of the technical replicates (3 biological replicates with at least 2 technical replicates each). *, *P* = 0.03, BT474 compared with other cells; *N* = 3 independent experiments. **D**. Gene copy number characteristics of breast cancer cell lines. **E**. Confocal images show the nucleation of TFO in different breast cancer cells. The red fluorescence indicates the TAMRA-tagged TFO fragment (Scale bar = 50 μm)

Firstly, we designed a TFO, HER2-11074, to target the region between exon 23 and exon 24 of the *HER2* gene, located at 39725858-39725883 (Fig. 1A). We selected breast cancer cells expressing different HER2 levels and detected and verified the HER2 expression (Fig. 1B-D). According to the Lip2000 reagent instructions, 800 ng of DNA was required for each well in24-well plates. The nucleation of TFO in different cells was observed by fluorescence confocal microscopy 24 h after transfection (Fig. 1E). TFO fragments entering different breast cancer cells can be seen successfully, and TFO is most abundant in the nucleus in HER2-overexpressing BT474 and least in HER2-non-expressing BT20 cells.

### Effects of TFO formation on cell proliferation and apoptosis

**Fig. 2:**
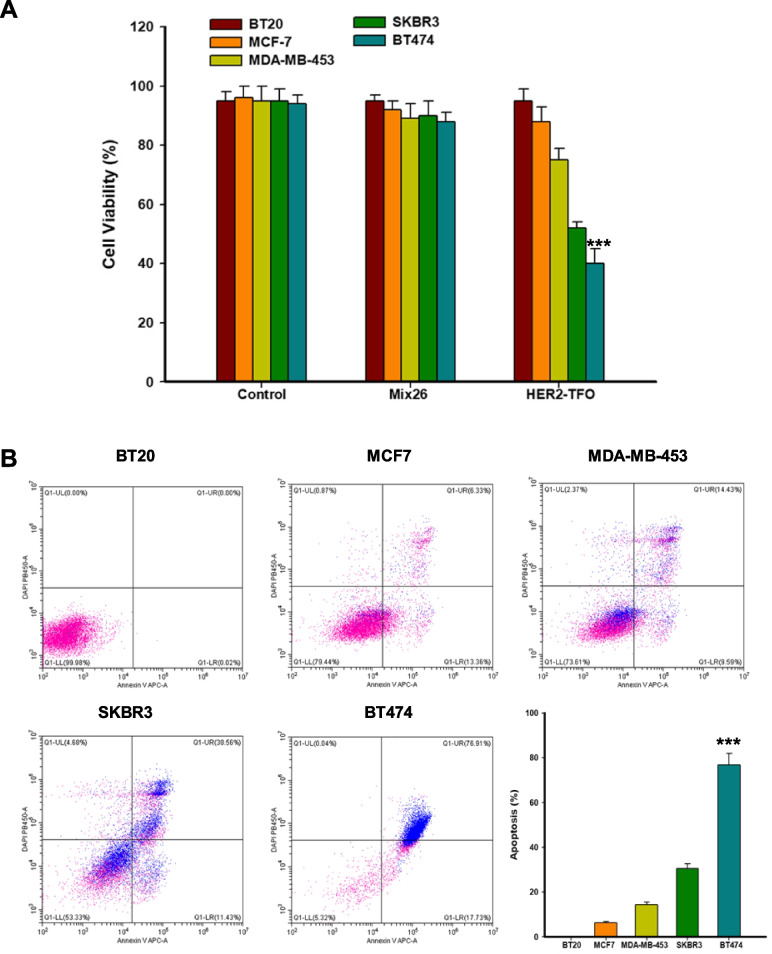
The effect of HER2-11074 on human HER2-positive cells. **A**. Cell viability was assessed using the CCK8 method after 72 h of treatment with HER2-targeting TFO-11074 in different breast cancer cells. Data are presented as mean ± SEM and were analyzed by Tukey's post hoc test; Error bars represent SEM of the technical replicates (3 biological replicates with at least 2 technical replicates each). ***, *P* = 0.0005; *N* = 3 independent experiments. **B**. Flow cytometry was used to analyze the transfection efficiency of TAMAR-TFO-11074 and its effect on apoptosis in different breast cancer cells. The blue dots represent signals from TAMAR-TFO-11074. The bar chart demonstrates the ratio of apoptosis. Results were analyzed by Tukey's post hoc test and the data are mean ± SEM (***, *P* = 0.001, BT474 compared with other cells)

CCK8 results showed that HER2-TFO significantly inhibited the growth of breast cancer tumor cells compared with control Mix26, especially in BT474 cells with high expression (Fig. 2A). The inhibition rate of cell growth was related to the expression level of HER2. Flow cytometry also showed that BT474 cells had the highest percentage of apoptosis. The blue markers were cells that had been successfully transferred into cells with TAMRA fluorescence. We could see that blue markers were most abundant among BT474 cells, and those with blue markers had the highest levels of apoptosis (Fig. 2B).

### Characterization of TFO@ZW-128

**Fig. 3:**
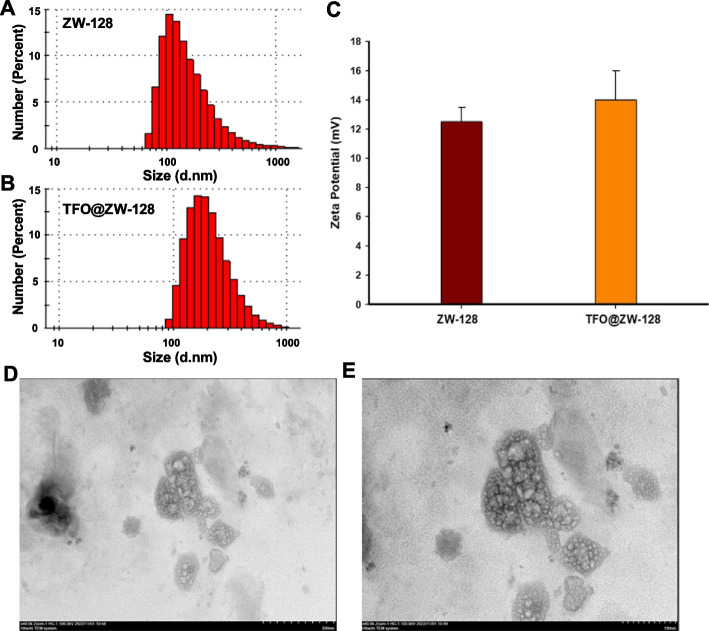
Characterization of TFO@ZW-128. **A**. The DLS of ZW-128. **B**. The DLS of TFO@ZW-128. **C**. Zeta potential of ZW-128 and TFO@ZW-128. Mean ± SD of three independent experiments. There was no statistical difference. **D**. The TEM of TFO@ZW-128 (200 nm). **E**. The TEM of TFO@ZW-128 (100 nm)

The DLS assay showed that the ZW-128 particle size distribution demonstrated a single narrow peak with an average particle size of 128 nm (Fig. 3A). The particle size distribution of TFO@ZW-128 was unimodal and normal, with an average particle size of 142 nm (Fig. 3B). ZW-128 and TFO@ZW-128 ZETA potentials were 12.5 mv and 13.5 mv, respectively (Fig. 3C). SEM images showed that TFO@ZW-128 was spherical in shape, with a smooth surface and uniform size. There was no adhesion between the nano-particles (Fig. 3D and E).

### In vitro therapy of TFO@ZW-128

**Fig. 4:**
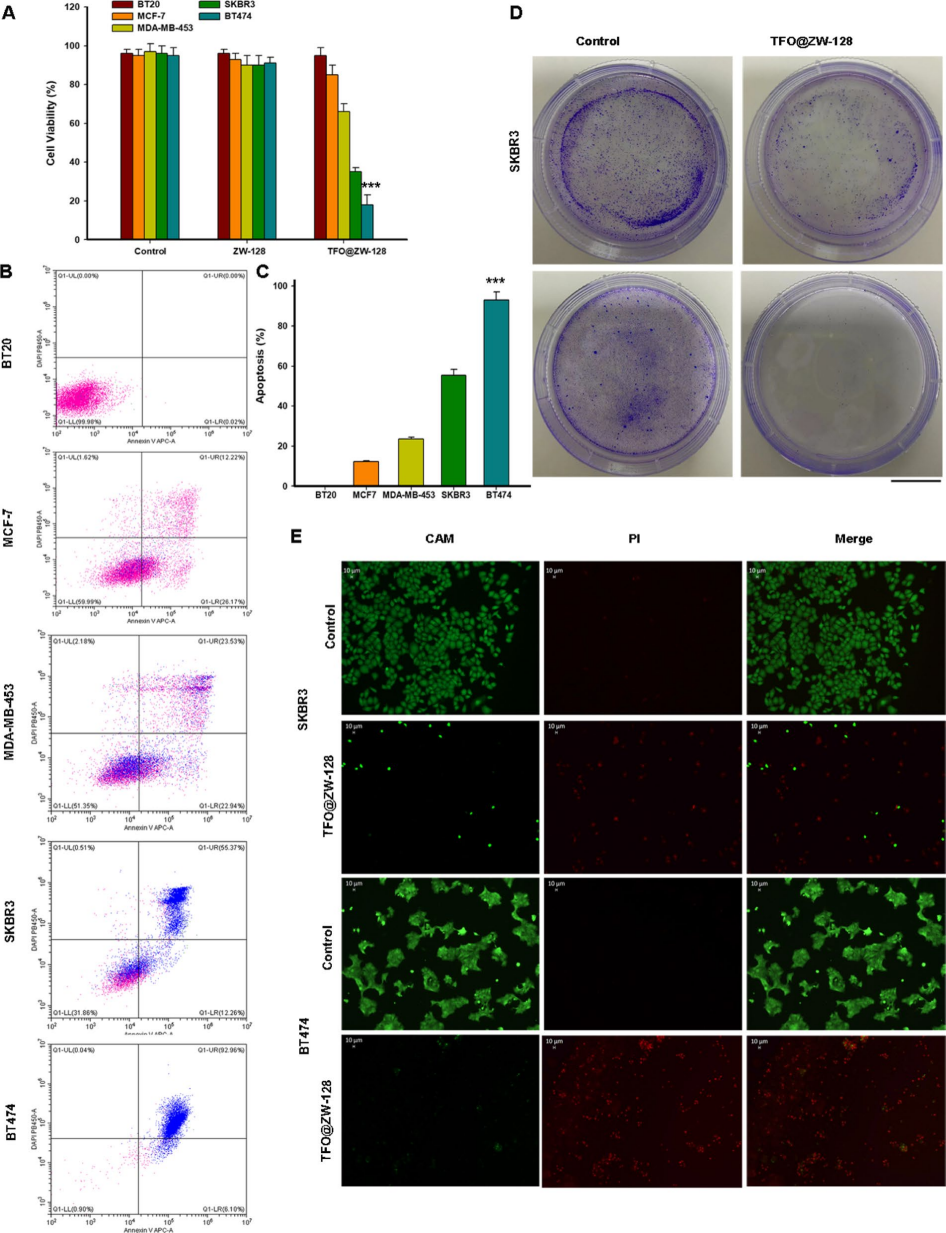
In vitro therapy of TFO@ZW-128. **A**. Cell viability was assessed using the CCK8 method after 72 h of treatment with TFO@ZW-128 in different breast cancer cells. Data are presented as mean ± SEM and were analyzed by paired Student’s t-test; Error bars represent SEM of the technical replicates (2 biological replicates with at least 2 technical replicates each). ***, *P* = 0.001; *N* = 3 independent experiments. **B**. Flow cytometry was used to analyze the cellular uptake of TFO@ZW-128 and its effect on apoptosis in different breast cancer cells. **C**. The bar chart demonstrates the ratio of apoptosis. Statistical significance was calculated by two-sided Student’s t test and the data are mean ± SEM (***, *P* = 0.008, BT474 compared with other cells). **D**. Clonogenic assays were performed after treating SKBR3 and BT474 cells with TFO@ZW-128. Scale bars: 10 mm. **E**. Live/dead staining of SKBR3 and BT474 cells treated with TFO@ZW-128. Scale bars: 10 μm

Firstly, the toxic effects of TFO@ZW-128 nano-particles on HER2-expressing tumor cells were studied by CCK-8. The results showed that TFO@ZW-128 had excellent biocompatibility and safety. It had low toxicity to tumor cells expressing no or low levels of HER2 (Fig. 4A) and high toxicity to cells with high HER2 expressing levels, with cell viability of BT474 cells decreasing to less than 20%. The incidence of apoptosis after TFO@ZW-128 treatment was studied by Flow cytometry. As shown in Fig. 4B and C, the apoptosis rate of BT474 cells treated with TFO@ZW-128 was 92.96%, which was much higher than that induced by Lip2000 transfection. In addition, the apoptosis rate of cells with high HER2 expression was significantly higher than that of cells with no or low HER2 expression. Clonogenic experiments confirmed that the number of cells in the TFO@ZW-128 group was significantly lower than that in the control group, and this trend was more pronounced in BT474 cells than in SKBR3 cells (Fig. 4D). Live/dead staining experiments were also performed to study tumor cell survival and to observe therapeutic effects on in vitro (Fig. 4E). Interestingly, cells in the control group fluoresced mostly green and barely at all red. However, the TFO@ZW-128 treated cells showed strong red fluorescence, which was more evident in BT474 cells than in SKBR3 cells.

### The anti-tumor mechanism of TFO@ZW-128 therapy

**Fig. 5:**
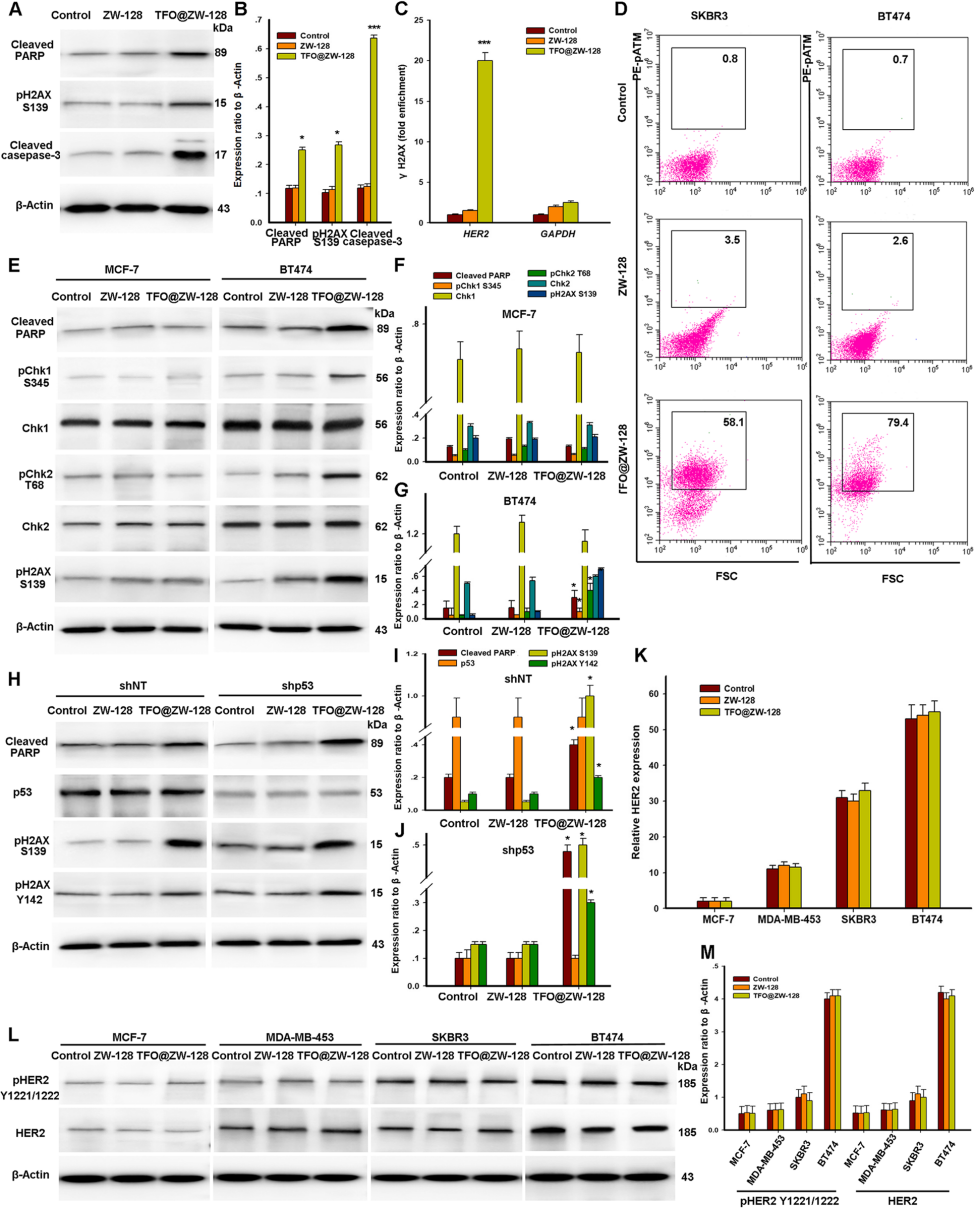
Mechanism of apoptosis induced by TFO@ZW-128. **A**. Western blot analysis of triplex-induced apoptosis as measured by cleaved PARP and DNA damage as measured by pH2AX S139 in BT474 cells 12 h following TFO@ZW-128 treatment. **B**. Quantification indicated the levels of cleaved PARP, pH2AX S139, and cleaved casepased-3 in these cells. Error bars represent SEM of the technical replicates (3 biological replicates with 3 technical replicates each). Statistical significance was calculated by Tukey's post hoc test. The data are mean ± SEM (*, *P* = 0.04; ***, *P* = 0.001, TFO@ZW-128-treated cells compared with other cells). **C**. ChIP analysis of BT474 cells demonstrating gene-specific enrichment of γH2AX at the HER2 target site and an absence of DNA damage at the non-targeted GAPDH locus 8 h after treatment with TFO@ZW-128. Data are presented as mean ± SEM and were analyzed by Tukey's post hoc test; ***, *P* = 0.001. **D**. Flow cytometry profiles of SKBR3 and BT474 cells stained for pATM. Cells were harvested 24 h after treatment. The box indicates the gate for high levels of pATM, and the numbers represent the percentage of cells with high levels of pATM. **E**. Western blot analysis of the phosphorylation status of the DNA damage response proteins Chk1 and Chk2 following TFO@ZW-128 treatment. **F**. Quantification indicated the above proteins levels in MCF7 cells. There was no statistical difference. **G**. Quantification indicated the above proteins levels in BT474 cells. Results of F and G were analyzed by Tukey's post hoc test and the data are mean ± SEM (*, *P* = 0.04, TFO@ZW-128-treated cells compared with other cells).**H**. TFO@ZW-128 activates p53-independent apoptosis in HER2-positive BT474 cells; shNT, short hairpin RNA (shRNA) against non-target control; shp53, shRNA against p53. **I**. Quantification indicated the above proteins levels in shNT cells. Results were analyzed by Tukey's post hoc test and the data are mean ± SEM (*, *P* = 0.03, TFO@ZW-128-treated cells compared with other cells). Error bars represent SEM of the technical replicates (2 biological replicates with 2 technical replicates each, *n* = 4). **J**. Quantification indicated the aboved proteins levels in shp53 cells. Results were analyzed by Tukey's post hoc test and the data are mean ± SEM (*, *P* = 0.04, TFO@ZW-128-treated cells compared with other cells). Error bars represent SEM of the technical replicates (2 biological replicates with 2 technical replicates each, *n* = 4) **K**. Analysis of *HER2* gene expression by RT-PCR (mean ± SD; Tukey's post hoc test; *N* = 3 independent experiments). **L**. Determination of HER2 protein levels and phosphorylation status by western blotting. **M**. Quantification indicated the above proteins levels in breast cancer cells. Results were analyzed by Tukey's post hoc test and the data are mean ± SEM. Error bars represent SEM of the technical replicates (2 biological replicates with 2 technical replicates each, *n* = 4)

Western blot analysis of Cleaved PARP confirmed that triplex-induced apoptosis was related to an increase in DSB, as shown by H2AX phosphorylation at S139 (Fig. 5A, B). To determine the TFO@ZW-128’s mechanism of action and characterize the DNA damage response activated in TFO@ZW-128-treated cells, we used chromatin immunoprecipitation (ChIP) of γH2AX to verify the target-specific induction of DNA damage by TFO@ZW-128. 8 h after TFO@ZW-128 treatment, we detected a 20-fold enrichment of γH2AX in the HER2 gene compared with those untreated cells (Fig. 5C). Furthermore, analysis of DNA damage induced by non-targeted regions of the genome using the GAPDH locus probe did not find γH2AX after TFO@ZW-128 treatment (Fig. 5C). These findings support that TFO-generated structures can specifically induce DNA damage at targeted amplified oncogenic loci.

We next checked the status of Chk1, Chk2, and ATM in HER2-positive cells after TFO@ZW-128 treatment. As shown in Fig. 5D, Chk1 phosphorylation at S345 was observed after treating HER2-amplified cells with TFO@ZW-128, whereas it was not observed in cells with normal *HER2* gene copy numbers. The activation of Chk1 in BT474 cells corresponded to the induction of DSB (pH2AX S139) and apoptosis (Cleaved PARP), respectively. In addition, triplet-induced phosphorylation of Chk2 at T68 by DSB was observed in BT474 cells (Fig. 5D). These phosphorylation events were related to an increase of phosphorylated ATM (pATM)-positive cells after TFO@ZW-128 treatment (Fig. 5E-G). The *p53* gene can regulate the pro-apoptotic pathway in severe DNA damage. Nevertheless, more than 50% of human cancers fail to trigger apoptosis due to loss of function caused by *p53* mutations. To test whether triplex-induced DNA damage could activate p53-independent apoptosis, we treated p53-depleted BT474 cells with TFO@ZW-128. We found that TFO treatment of p53-depleted cells resulted in similar levels of PARP cleavage compared with the treatment of cells in the control group, confirming that triple-strand formation can activate apoptosis regardless of *p53* status (Fig. 5H-J). Triplex-induced DSB induced robust H2AX Y142 phosphorylation without p53 (Fig. 5H-J).

To further demonstrate the relationship between triad formation and the cellular function of *HER2*, we analyzed the expression of the *HER2* gene by RT-PCR (Fig. 5K). We also detected total HER2 protein and phosphorylation levels in some breast cancer cell lines (Fig. 5G). Our results showed that HER2 gene expression was not significantly affected by TFO@ZW-128 treatment (Fig. 5L). Moreover, compared with cells in the control group, total and activated HER2 levels remained the same after the triad-induced apoptosis of HER2-positive cells (Fig. 5M). Taken together, these results indicate that the function of TFO targeting HER2 is independent of HER2 cell function.

### Anti-cancer efficacy and safety of TFO@ZW-128 in vivo

**Fig. 6:**
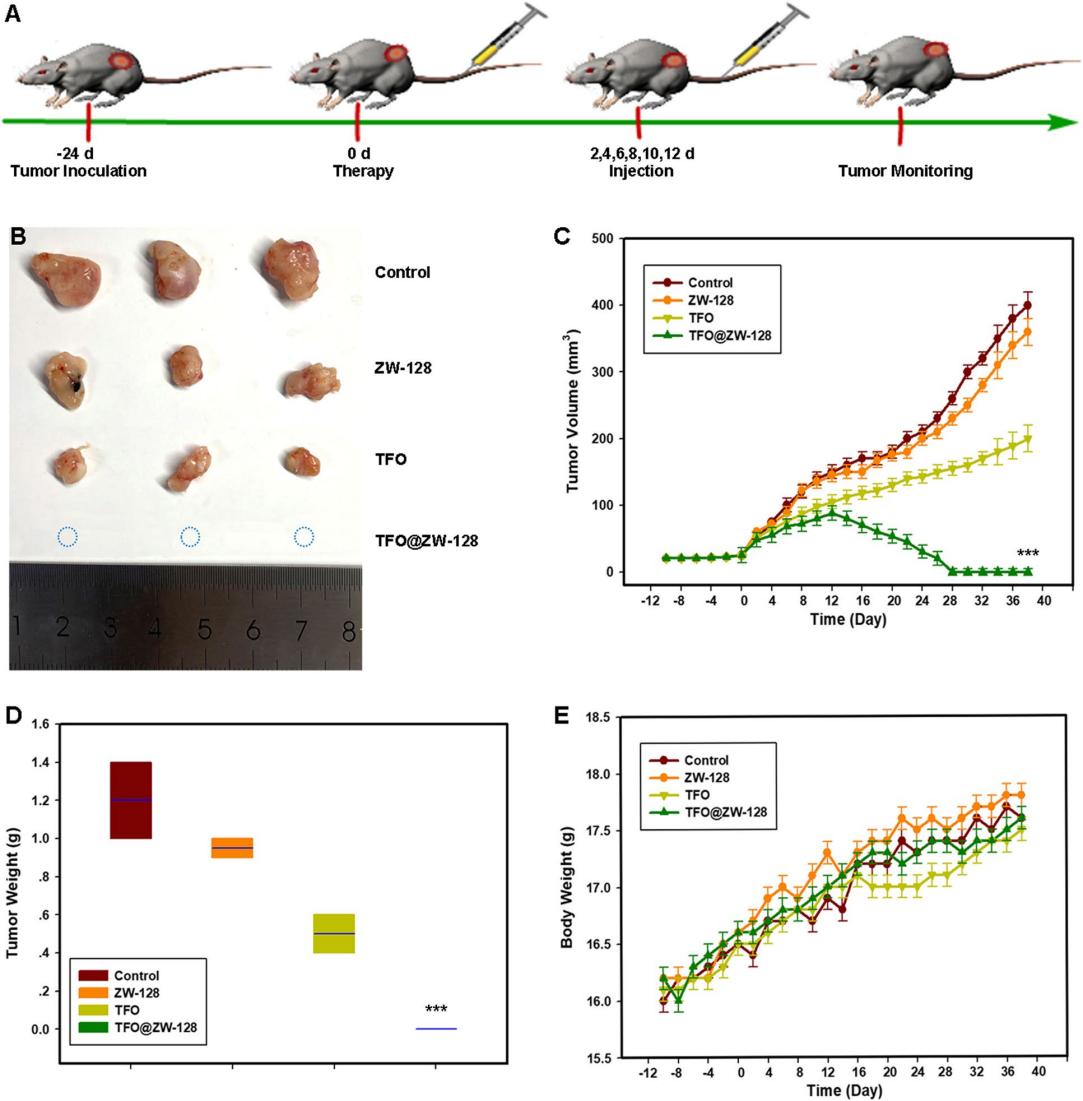
Antitumor effects of TFO@ZW-128 in the BT474 tumor model. **A**. Schematic illustration of establishing BT474 tumor-bearing mice model for anti-tumor effect. **B**. The tumor photo of the tumor. **C**. The tumor volume growth curves of tumors. Results were analyzed by Tukey's post hoc test and the data are mean ± SEM (***, *P* = 0.001, TFO@ZW-128-treated compared with others). Error bars represent SEM of the technical replicates (*n* = 3). **D**. The weight of the tumor. Results were analyzed by Tukey's post hoc test and the data are mean ± SEM (***, *P* = 0.001, TFO@ZW-128-treated compared with others). Error bars represent SEM of the technical replicates (*n* = 3). **E**. Body weight change curves in different groups. There was no statistical difference

The anti-tumor effect of TFO@ZW-128 in vivo was determined in BALB/c female nude mice bearing BT474 cell xenografts. 24 days after subcutaneous injection of tumor cells, mice were injected every two days with TFO@ZW-128 (200ul, with 5ug TFO per 20 g included, iv), ZW-128(200ul, 140ug/20 g, iv) or 0.9% saline (200ul, iv); TFO was administered intraperitoneally on days 0, 3, and 7 at a dose of 200 ul and 400 ug/20 g (Fig. 6A). The tumor size and body weight of mice were continuously measured 2 weeks after tumor inoculation until the end of the experiment. Our results showed that TFO@ZW-128 performed better than TFO, ZW-128, and saline in inhibiting tumor growth. The dosage of TFO@ZW-128 is 80 times smaller than that of TFO. ZW-128 is safe and improves the utilization rate of TFO fragments. The results showed that TFO@ZW-128 nano-particles ensured that HER2-positive breast cancer cells took up more TFO. There was no tumor regression in the ZW-128 and saline groups (Fig. 6B-D). There was no difference in body weight among the four groups (Fig. 6E).

**Fig. 7:**
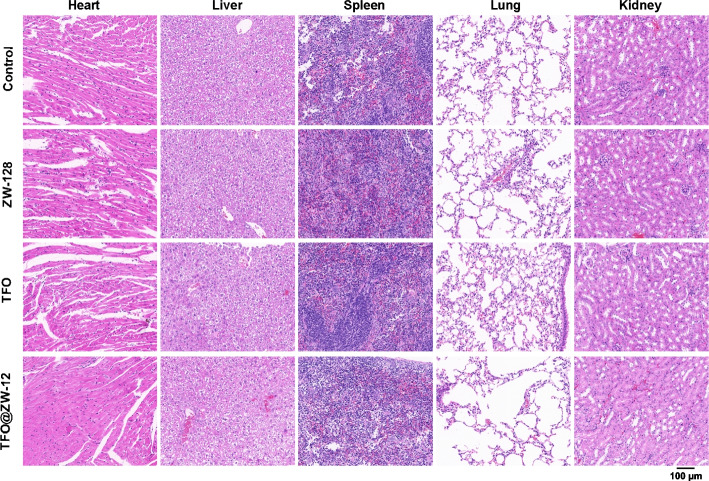
The safety of TFO@ZW-128 in vivo. Major organs (heart, liver, spleen, lung, and kidney) from the tumor-bearing mice in different treatment groups with H&E staining after different treatments (Scale bar = 100 μm)

To further confirm the potential toxicity of TFO@ZW-128 in vivo, H & E staining was used for the pathological analysis of the main organs. As shown in Fig. 7, after 14 days of treatment and 24 days of observation, no tissue damage or inflammation occurred in the major organs, including the heart, liver, spleen, lungs, and kidneys. The results showed that TFO@ZW-128, a nano-drug, was highly biocompatible. All these studies systematically confirm the application of TFO@ZW-128 in treating tumors with high expression of HER2 in vivo.

## Discussion

As a new biomaterial, amino acid polymers have the following advantages [17]: 1. It is naturally stable and resistant to organic solvents. 2. The degradation products are primarily amino acids, lactic acid, water, and carbon dioxide, which are non-toxic with no damage to the patient body. 3. The main chain of the polymer has amino groups on both sides, which provides binding sites, and can be easily polymerized with other materials to obtain a variety of modified copolymers for different use. 4. It has good compatibility and affinity and can be degraded in vivo. The degradation rate is adjustable. Due to these characteristics, amino acid polymers can compensate for the shortcomings of other biodegradable materials. For example, polylactic acid is not strong enough and has slow degradation; moreover, its acid products cannot make for killing tumor cells. The degradation of inorganic salts is too fast or too slow. Amino acid polymers used as drug carriers for clinical application are gradually becoming a research hotspot [18–20].

TFO binds to double-stranded DNA in a sequence-specific manner to form a local triple-stranded helical structure, which can inhibit protein binding at the promoter region, block the extension of transcription, and induce DNA mutation and recombination [21]. Since TFO is a linear hydrophilic nucleic acid molecule, and the cell membrane is a lipid-soluble bilayer membrane with no nucleic acid channel, it is difficult for naked TFO to go through the cell membrane [11]. Tum cells' uptake rate of TFO increased significantly after encapsulation of ZW-128 nano-particles. The potential reason could be that the polymer nano-particles can enter the cells by endocytosis [22]. In addition, since polymer nano-particle has extremely small size and high surface energy, nano-particles can penetrate directly into the cell membrane when they have appropriate properties [23–25], resulting in a higher growth inhibitory effect of TFO@ZW-128 than that of naked TFO. Particle size and drug loading are essential indices to evaluate nano-particle quality [26, 27]. The ZW-128 used in this study was homogeneous and spherical, with an average particle size of 128 nm comparable to similar studies. Even though the preparation protocol for ZW-128 could not be released publicly due to patent issues, the findings strongly implied that the preparation method for ZW-128 is effective.

We demonstrated that triad formation could activate p53-independent apoptosis and that induction of DNA damage response by TFO treatment is effective. Treating HER2-positive breast cancer xenografts with TFO@ZW-128 resulted in a 50% reduction in tumor volume compared with untreated cells and an 80-fold reduction in usage compared with naked TFO. These results lay a foundation for targeted therapy of tumors with high expression of the *HER2* gene. Our research has some limitations. First of all, the major drawback is that we can't get the method to make TFO@ZW-128 because of patent restrictions. Moreover, we did not validate the safety and efficacy of TFO@ZW-128 in other HER2-positive cancer, such as gastric cancer and ovarian cancer. Future study will further elucidate the anti-cancer function of TFO@ZW-128 in HER2-positive cancers.

## Conclusion

The results showed that TFO@ZW-128 nano-particles had a good effect on HER2-overexpressing tumors, and the tumor volume of BT474-bearing nude mice was significantly reduced. TFO@ZW -128 has significant targeting activity in vivo and in vitro. It can effectively inhibit tumor growth, improve the drug utilization rate and contribute to the therapeutic effect of inhibiting tumor growth. Further research will understand the molecular composition of ZW-128 in detail and complete acute toxicological studies in animals. To sum up, TFO@ZW-128 plays an essential role in treating HER2-overexpressing tumors and provides a new perspective for tumor-targeted therapy.

## Supplementary Information

**Additional file 1.**

## Data Availability

All data generated or analyzed during this study are included in this published article.

## References

[1] LaBoy C, Siziopikou KP, Rosen L, Blanco LZ, Pincus JL (2021). Clinicopathologic features of unexpectedly HER2 positive breast carcinomas: An institutional experience. Pathol Res Pract.

[2] Vranić S, Bešlija S, Gatalica Z (2021). Targeting HER2 expression in cancer: New drugs and new indications. Bosn J Basic Med Sci.

[3] Pernas S, Tolaney SM (2022). Clinical trial data and emerging strategies: HER2-positive breast cancer. Breast Cancer Res Treat.

[4] Yang J, Ju J, Guo L, Ji B, Shi S, Yang Z, Gao S, Yuan X, Tian G, Liang Y (2022). Prediction of HER2-positive breast cancer recurrence and metastasis risk from histopathological images and clinical information via multimodal deep learning. Comput Struct Biotechnol J.

[5] Boulware SB, Christensen LA, Thames H, Coghlan L, Vasquez KM, Finch RA (2014). Triplex-forming oligonucleotides targeting c-MYC potentiate the anti-tumor activity of gemcitabine in a mouse model of human cancer. Mol Carcinog.

[6] Zuin Fantoni N, McGorman B, Molphy Z, Singleton D, Walsh S, El-Sagheer AH, McKee V, Brown T, Kellett A (2020). Development of Gene-Targeted Polypyridyl Triplex-Forming Oligonucleotide Hybrids. ChemBioChem.

[7] Economos NG, Thapar U, Balasubramanian N, Karras GI, Glazer PM (2022). An ELISA-based platform for rapid identification of structure-dependent nucleic acid-protein interactions detects novel DNA triplex interactors. J Biol Chem.

[8] Rogers FA, Tiwari MK (2013). Triplex-induced DNA damage response. Yale J Biol Med.

[9] Rogers FA, Vasquez KM, Egholm M, Glazer PM (2002). Site-directed recombination via bifunctional PNA-DNA conjugates. Proc Natl Acad Sci USA.

[10] Kaushik Tiwari M, Colon-Rios DA, Tumu HCR, Liu Y, Quijano E, Krysztofiak A, Chan C, Song E, Braddock DT, Suh HW (2022). Direct targeting of amplified gene loci for proapoptotic anticancer therapy. Nat Biotechnol.

[11] Jiao J, Zou Q, Zou MH, Guo RM, Zhu S, Zhang Y (2016). Aptamer-modified PLGA nanoparticle delivery of triplex forming oligonucleotide for targeted prostate cancer therapy. Neoplasma.

[12] Awasthi R, Roseblade A, Hansbro PM, Rathbone MJ, Dua K, Bebawy M (2018). Nanoparticles in Cancer Treatment: Opportunities and Obstacles. Curr Drug Targets.

[13] Cai L, Liu S, Guo J, Jia YG (2020). Polypeptide-based self-healing hydrogels: Design and biomedical applications. Acta Biomater.

[14] Ji Y, Song W, Xu L, Yu DG, Annie Bligh SW (2022). A review on Electrospun Poly(amino acid) nanofibers and their applications of hemostasis and wound healing. Biomolecules.

[15] Chen L, Wang B, Ren H, Wu Y, Lyu D, Ouyang Y, Zhang Q, Yan Y (2022). Arg-Gly-Asp peptide functionalized poly-amino acid/ poly (p-benzamide) copolymer with enhanced mechanical properties and osteogenicity. Biomater Adv.

[16] Barreto C, Jandus A (2022). Role of Natural Products in Combating Cancer. Cancer Insight.

[17] Thompson M, Scholz C (2021). Highly branched polymers based on poly(amino acid)s for biomedical application. Nanomaterials (Basel).

[18] Ansari V, Calore A, Zonderland J, Harings JAW, Moroni L, Bernaerts KV (2022). Additive Manufacturing of alpha-Amino Acid Based Poly(ester amide)s for Biomedical Applications. Biomacromol.

[19] Li C, You X, Xu X, Wu B, Liu Y, Tong T, Chen J, Li Y, Dai C, Ye Z (2022). A Metabolic Reprogramming Amino Acid Polymer as an Immunosurveillance Activator and Leukemia Targeting Drug Carrier for T-Cell Acute Lymphoblastic Leukemia. Adv Sci (Weinh).

[20] Gupta SS, Mishra V, Mukherjee MD, Saini P, Ranjan KR (2021). Amino acid derived biopolymers: Recent advances and biomedical applications. Int J Biol Macromol.

[21] Ohkubo A, Ohnishi T, Nishizawa S, Nishimura Y, Hisamatsu S (2020). The ability of a triplex-forming oligonucleotide to recognize T-A and C-G base pairs in a DNA duplex is enhanced by incorporating N-acetyl-2,7-diaminoquinoline. Bioorg Med Chem.

[22] Xu D, Ma Y, Han X, Chen Y (2021). Systematic toxicity evaluation of polystyrene nanoplastics on mice and molecular mechanism investigation about their internalization into Caco-2 cells. J Hazard Mater.

[23] Sarasamma S, Audira G, Siregar P, Malhotra N, Lai YH, Liang ST (2020). Nanoplastics cause neurobehavioral impairments, reproductive and oxidative damages, and biomarker responses in Zebrafish: throwing up alarms of wide spread health risk of exposure. Int J Mol Sci.

[24] Gupta J, Safdari HA, Hoque M (2021). Nanoparticle mediated cancer immunotherapy. Semin Cancer Biol.

[25] Hussein S, Ruben J (2022). Drug Transport via Nanocarrier for Liver Cancer Treatment. Cancer Insight.

[26] Oberoi HS, Arce F, Purohit HS, Yu M, Fowler CA, Zhou D, Law D (2023). Design of a Re-Dispersible High Drug Load Amorphous Formulation. J Pharm Sci.

[27] Han G, Xiang S, Jiang K, Zhang W, Weng Q (2023). Design of size uniform and controllable covalent organic framework nanoparticles for high-performance anticancer drug delivery. J Biomater Appl.

