# Supplementary material for: Nanoparticle delivery of TFOs is a novel targeted therapy for HER2 amplified breast cancer
Source: BMC Cancer. 2023 Jul 20;23:680. doi: 10.1186/s12885-023-11176-8 (PMC10357592; doi:10.1186/s12885-023-11176-8)
Supplement: Supplementary file 1 — Additional file 1. [file 12885_2023_11176_MOESM1_ESM.pptx]

## Slide 1
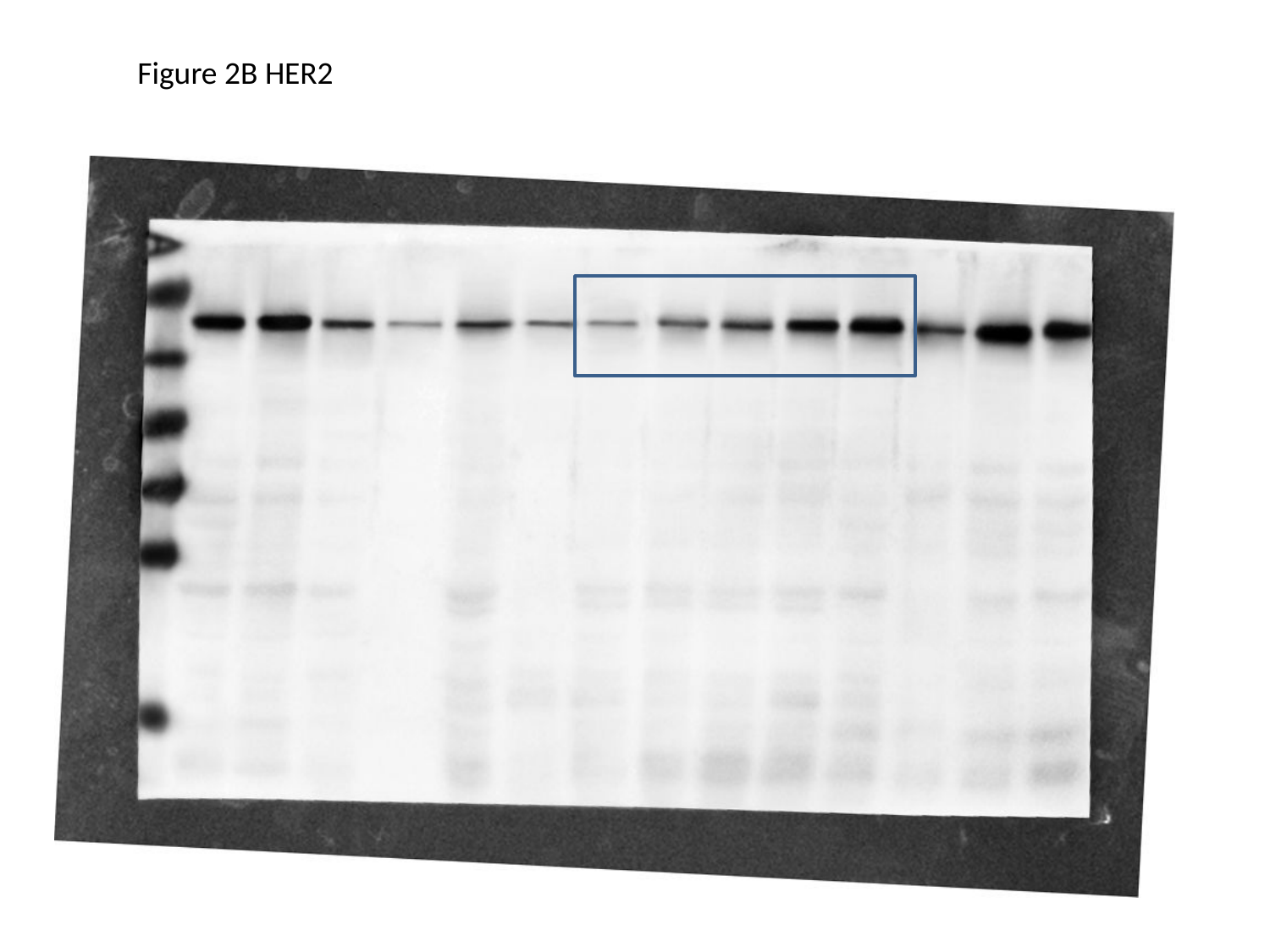

Figure 2B HER2

## Slide 2
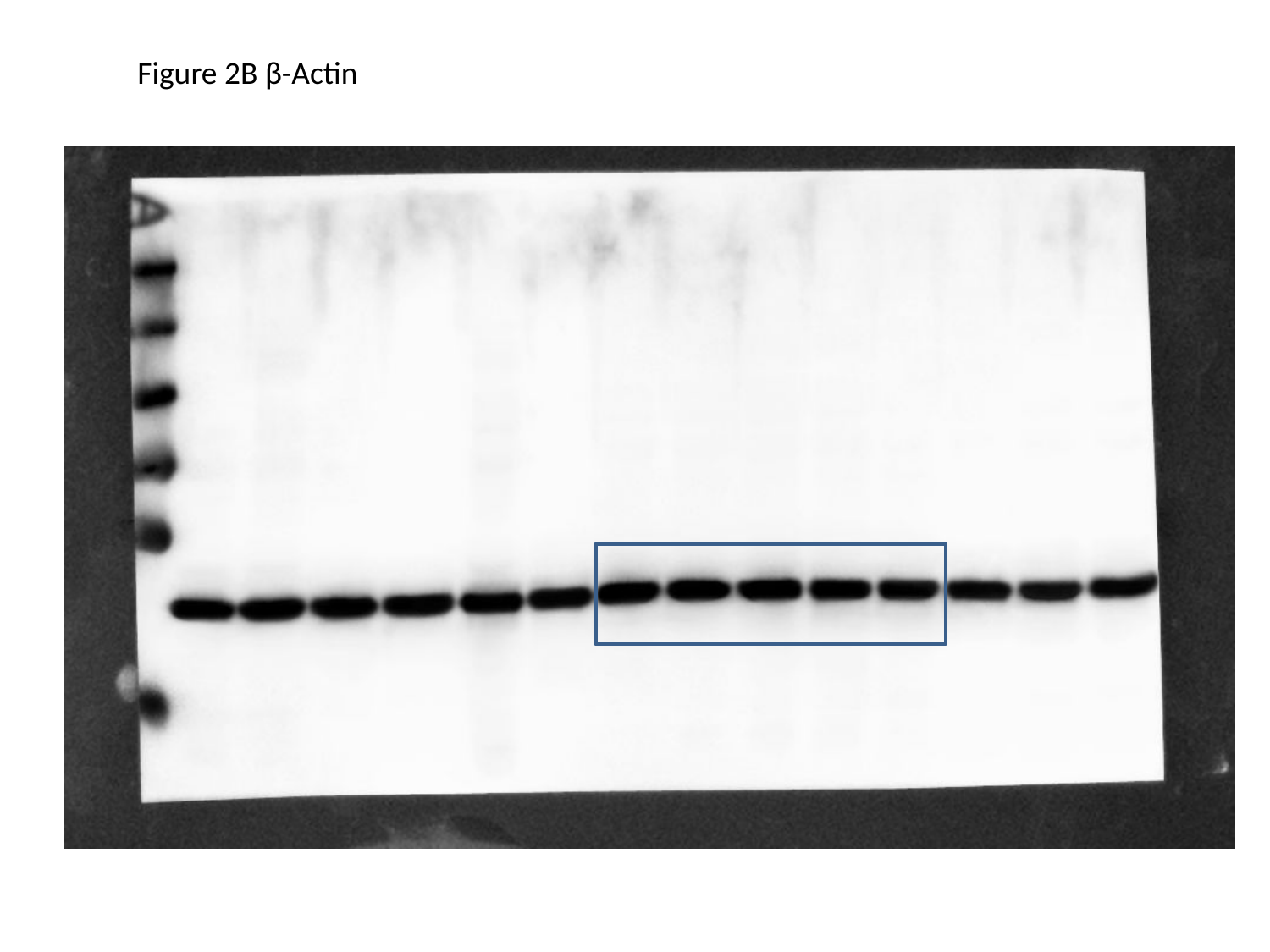

Figure 2B β-Actin

## Slide 3
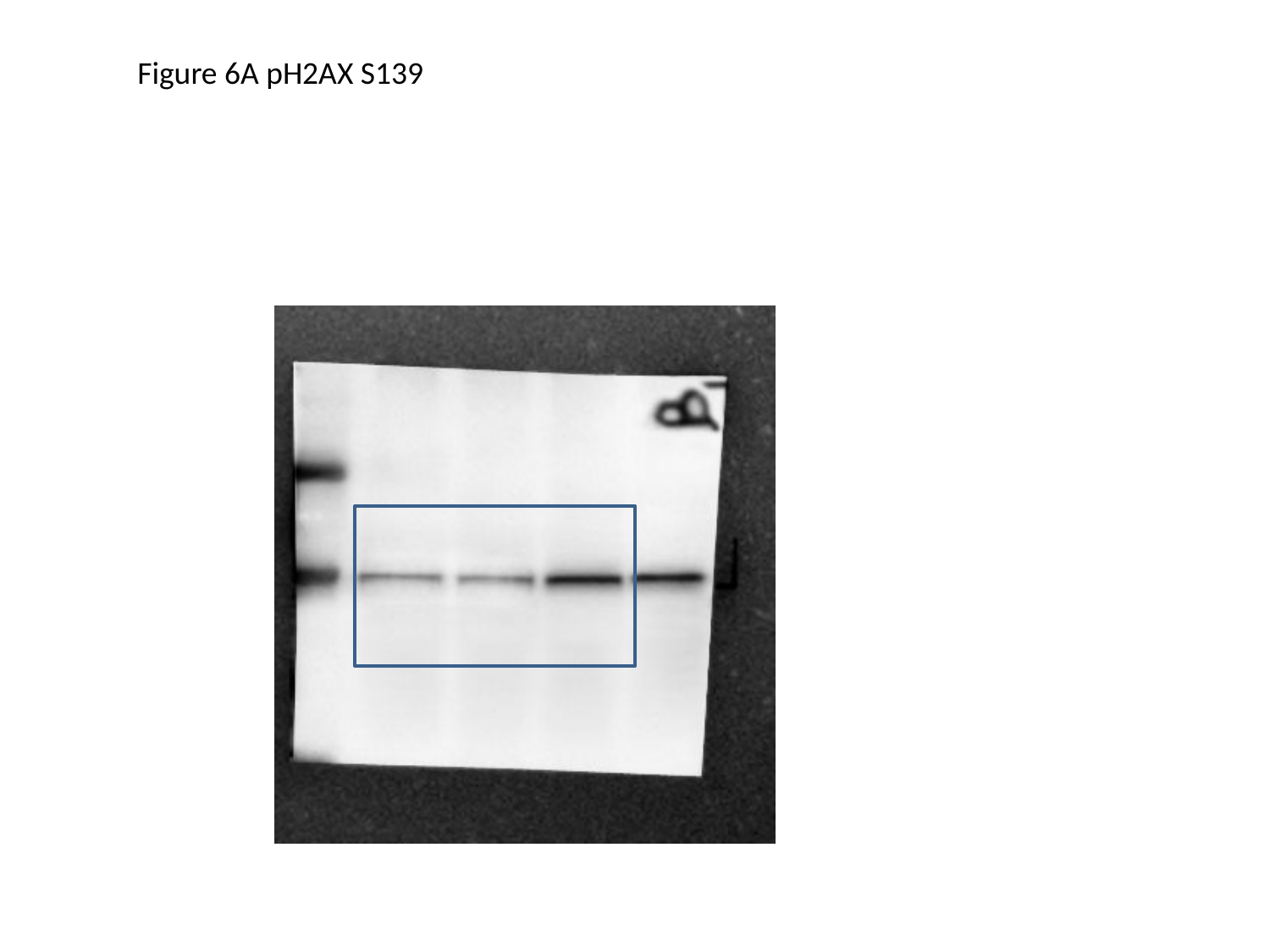

Figure 6A pH2AX S139

## Slide 4
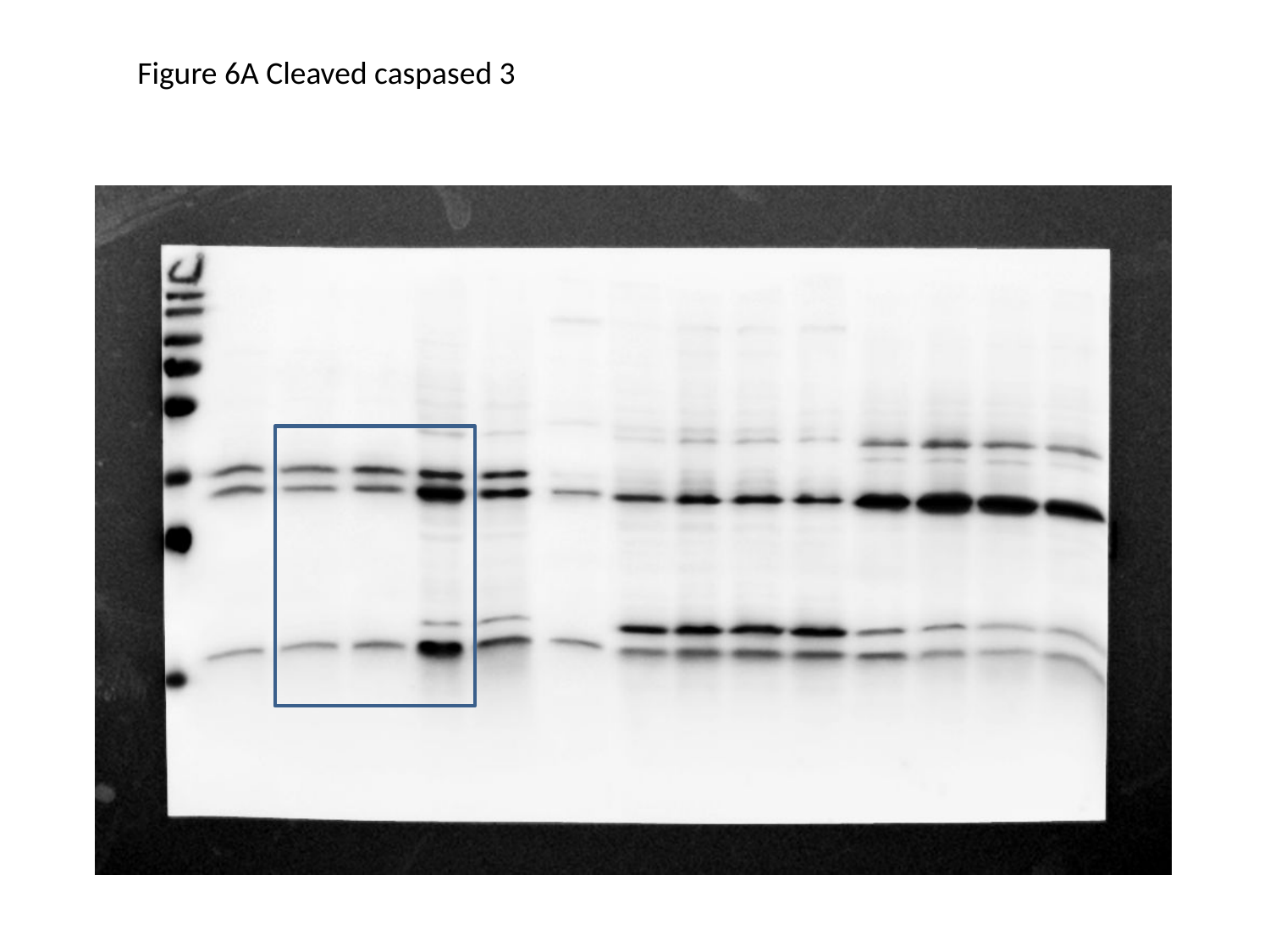

Figure 6A Cleaved caspased 3

## Slide 5
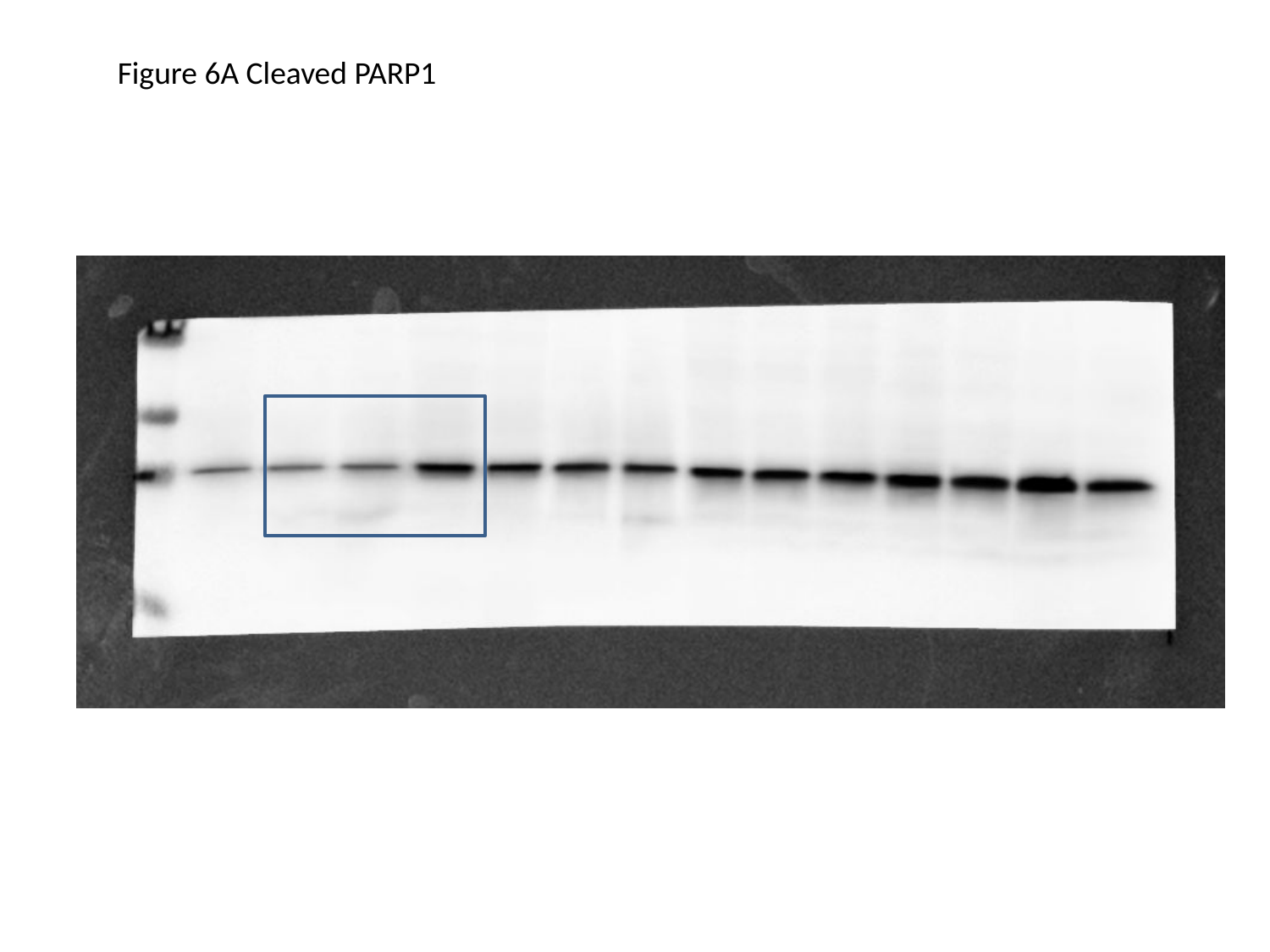

Figure 6A Cleaved PARP1

## Slide 6
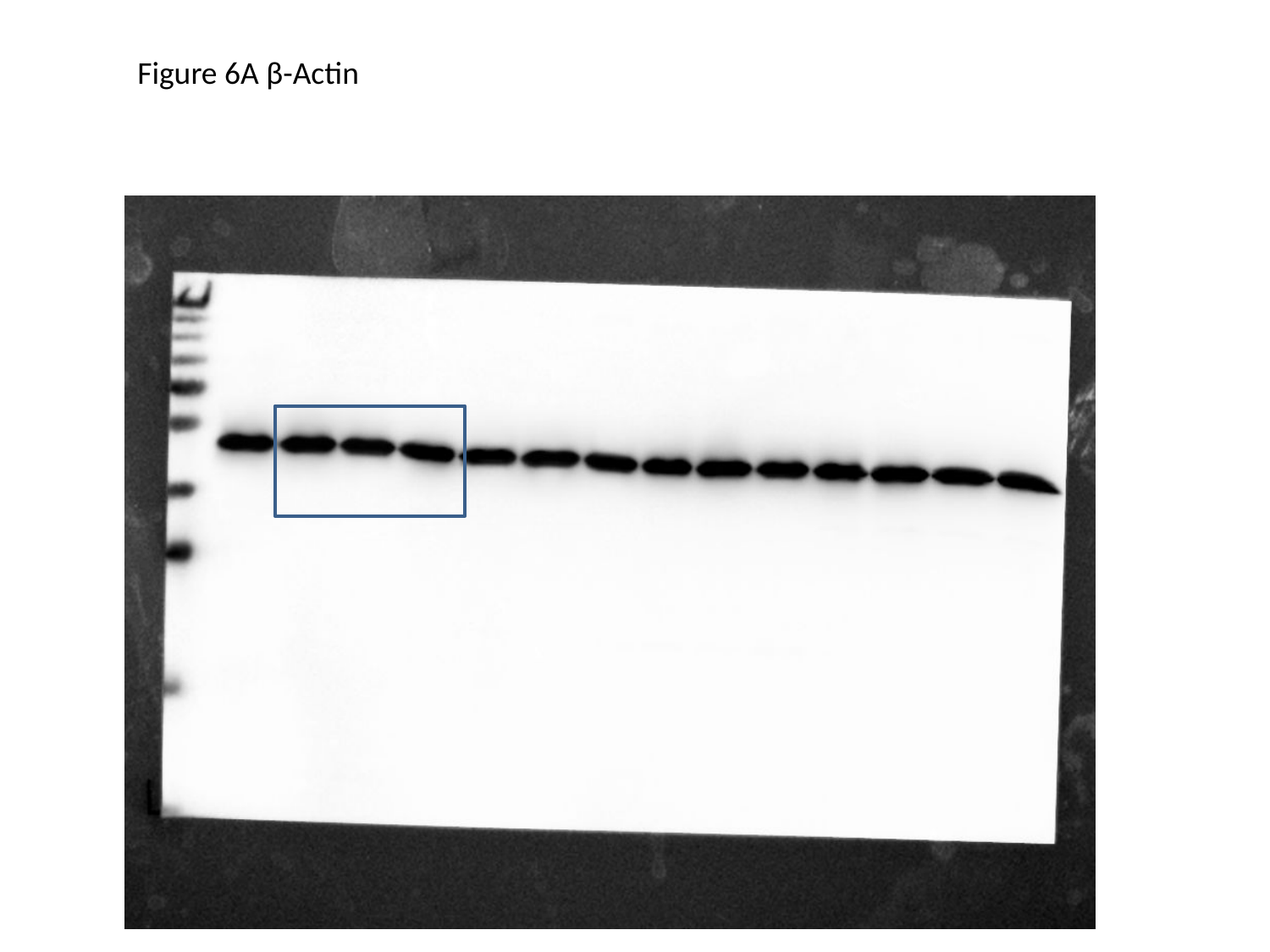

Figure 6A β-Actin

## Slide 7
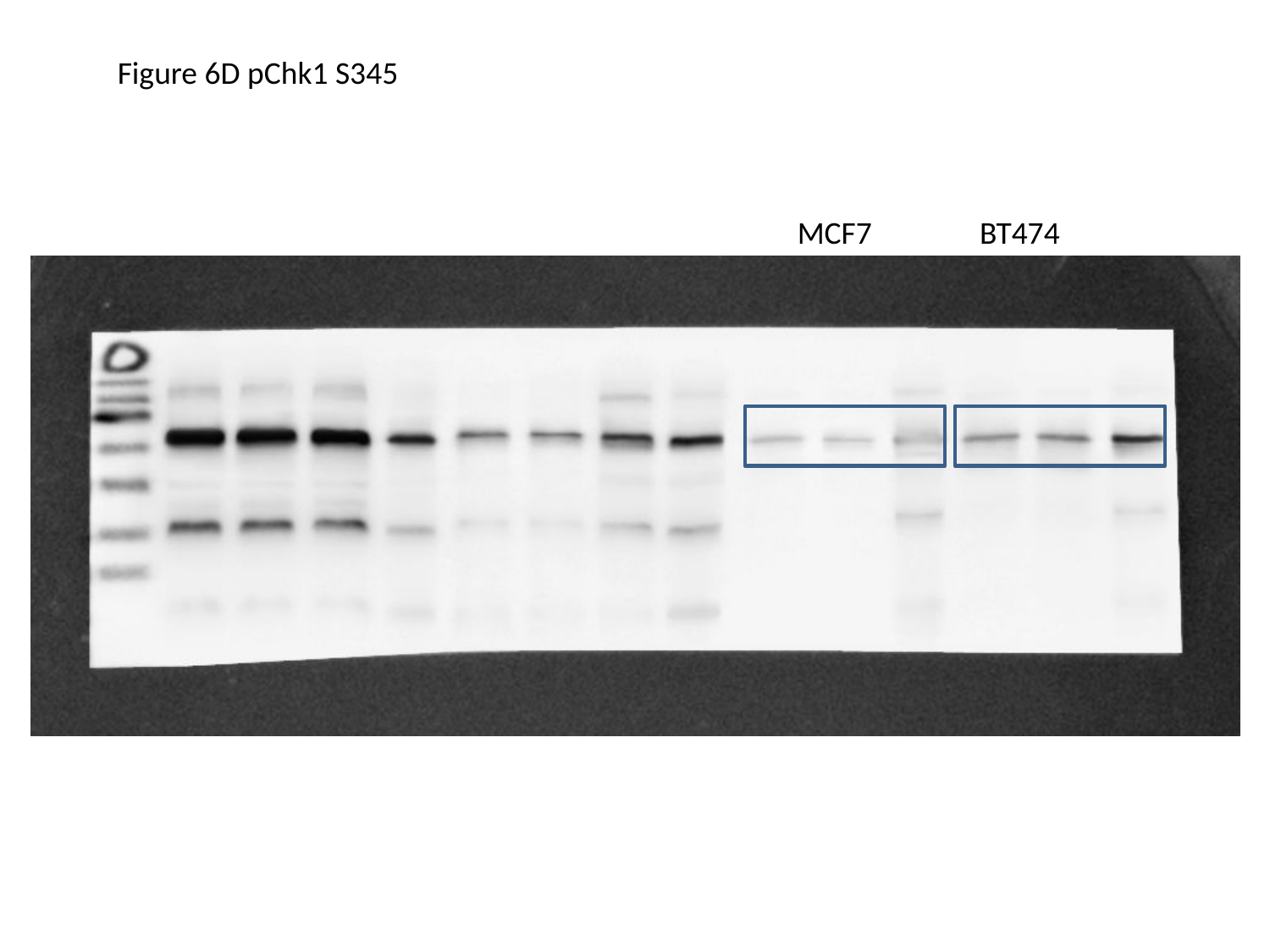

Figure 6D pChk1 S345
MCF7 BT474

## Slide 8
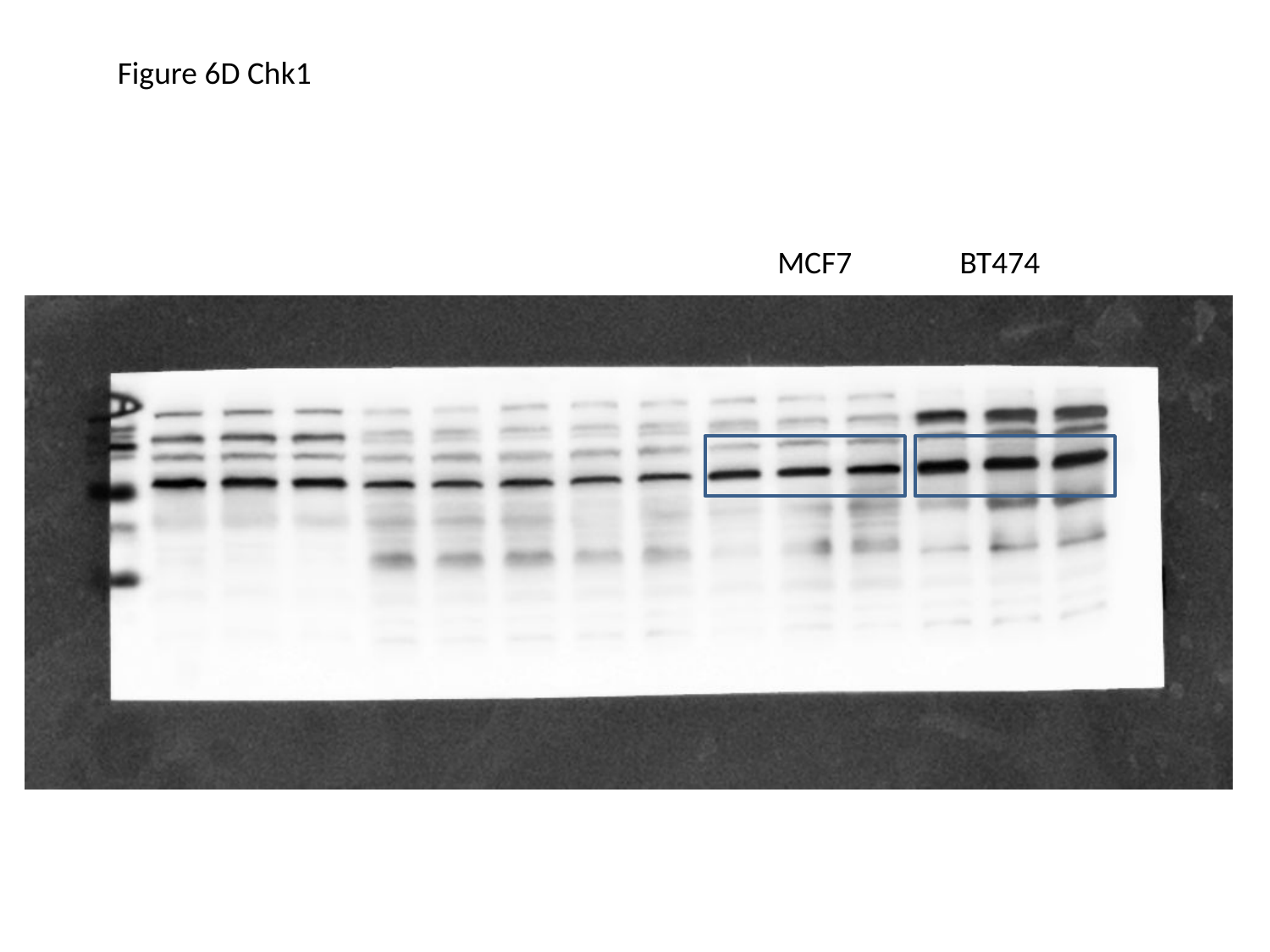

Figure 6D Chk1
MCF7 BT474

## Slide 9
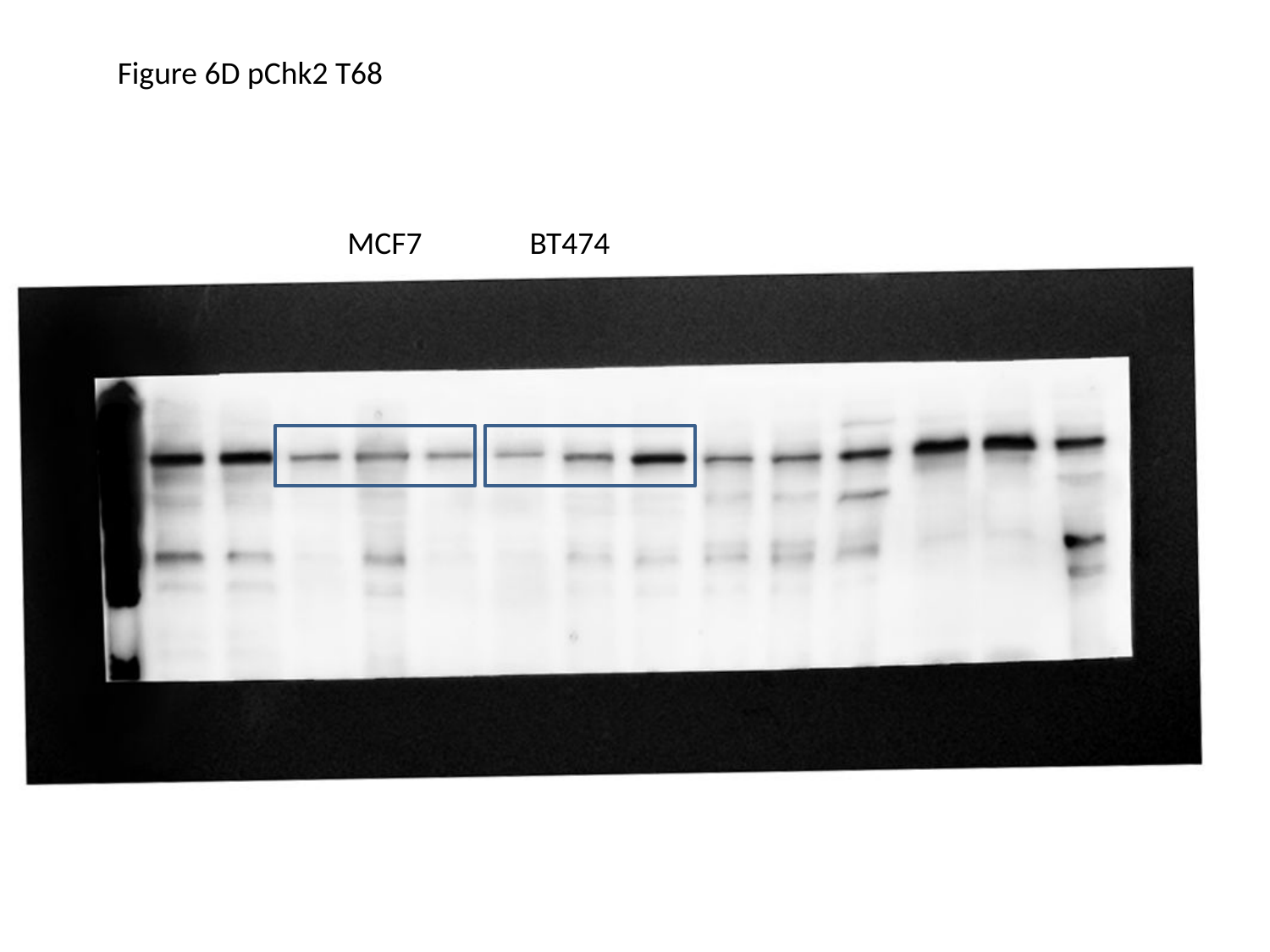

Figure 6D pChk2 T68
MCF7 BT474

## Slide 10
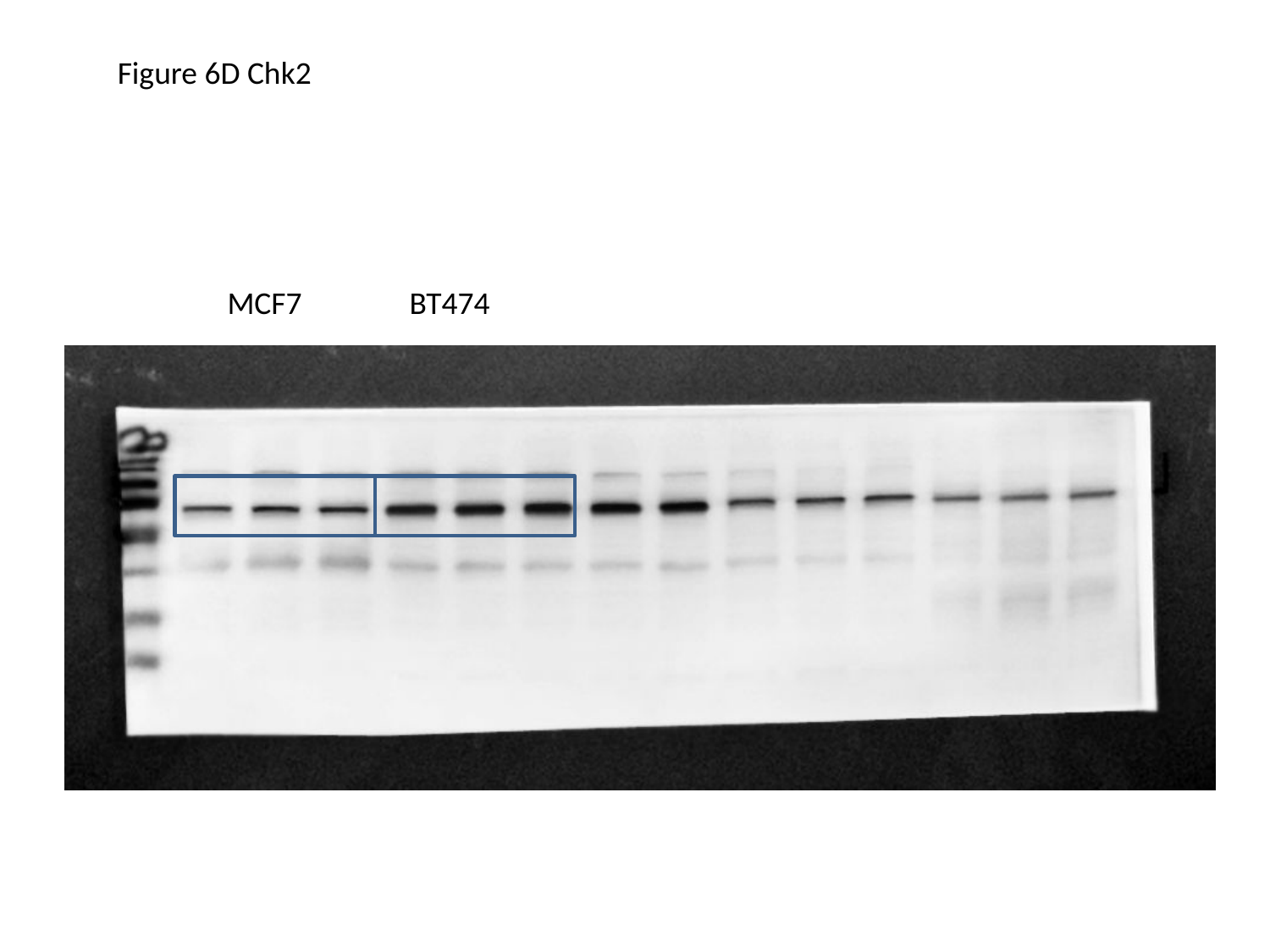

Figure 6D Chk2
MCF7 BT474

## Slide 11
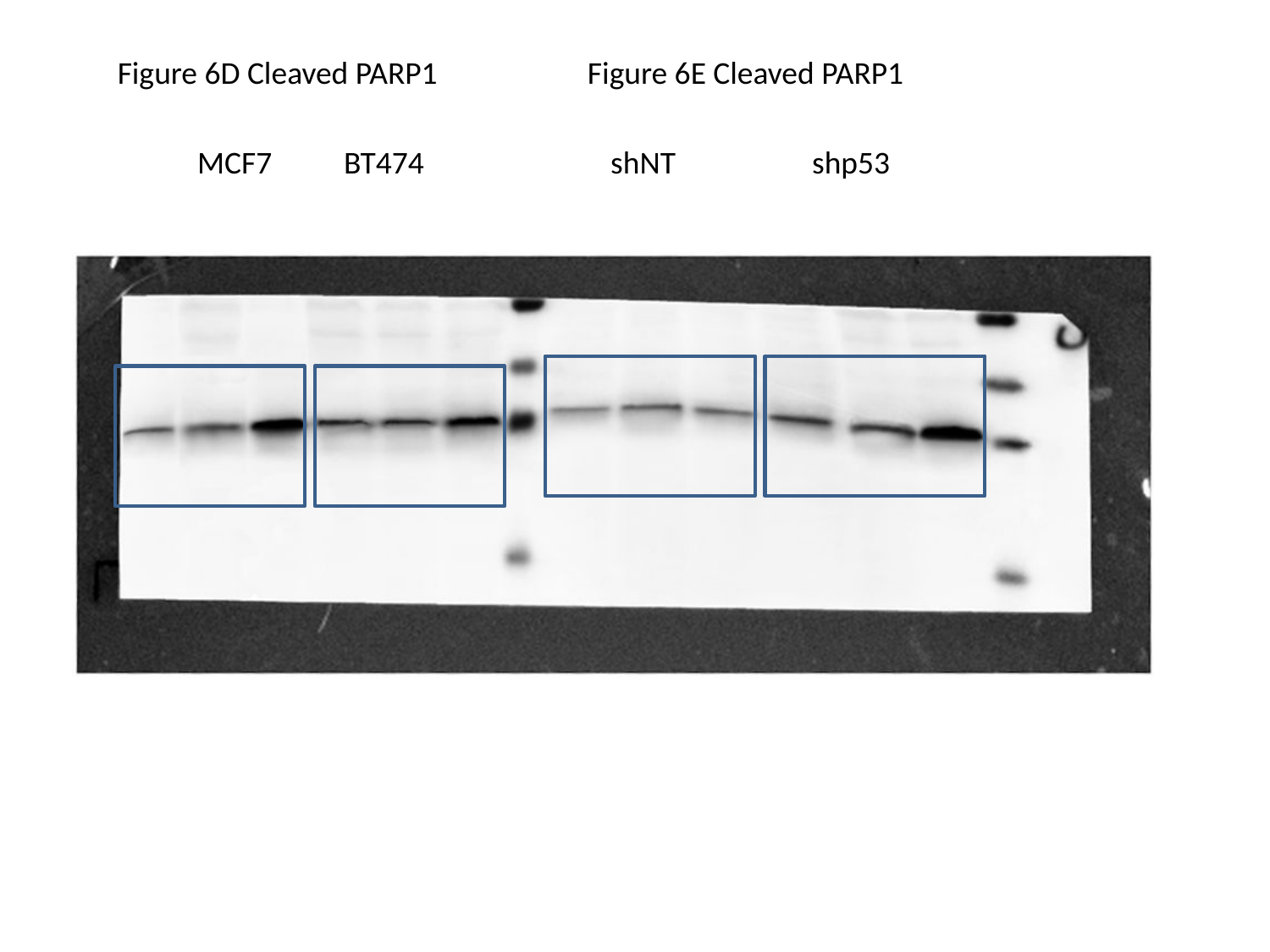

Figure 6D Cleaved PARP1
Figure 6E Cleaved PARP1
MCF7 BT474 shNT shp53

## Slide 12
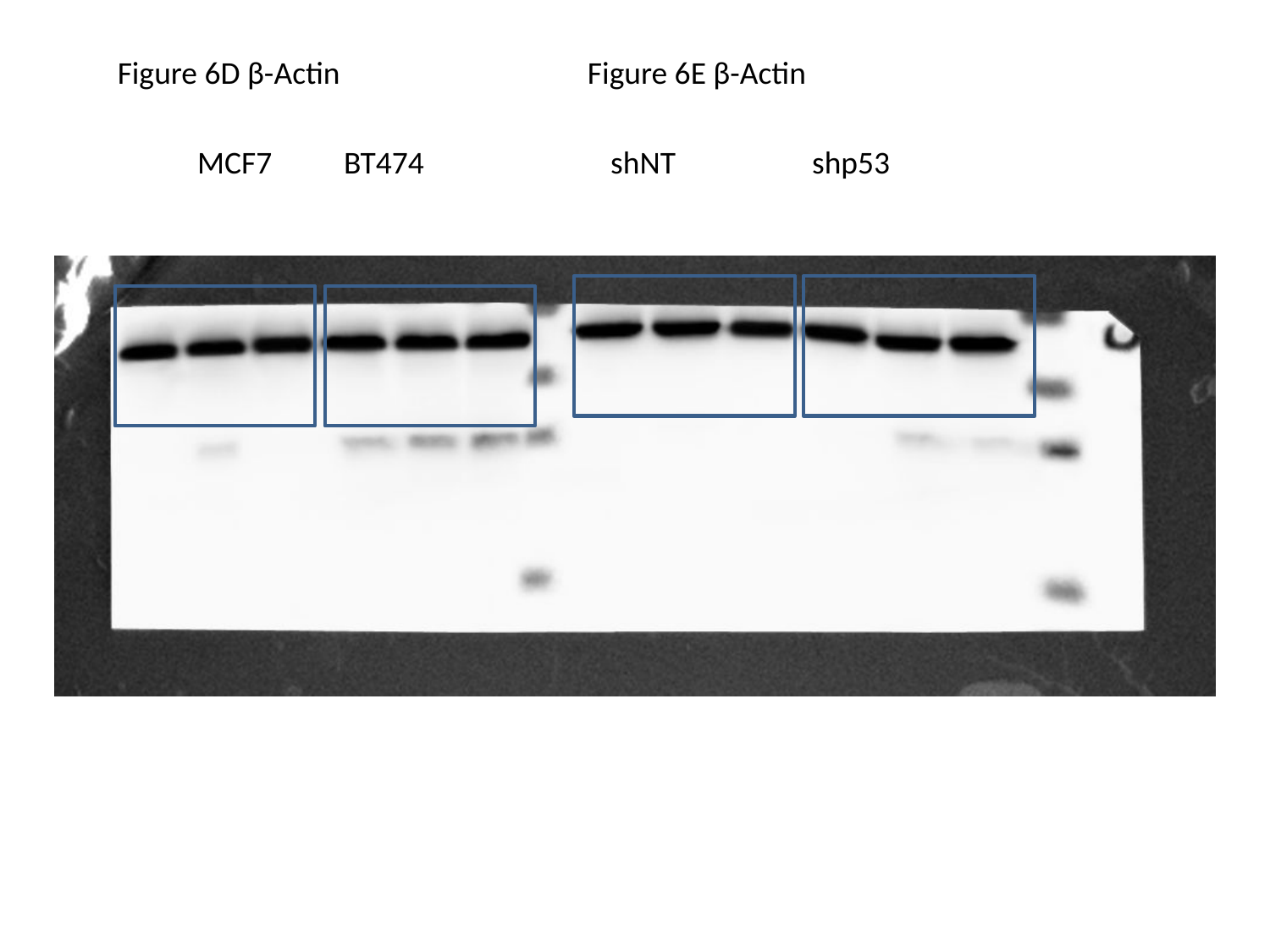

Figure 6D β-Actin
Figure 6E β-Actin
MCF7 BT474 shNT shp53

## Slide 13
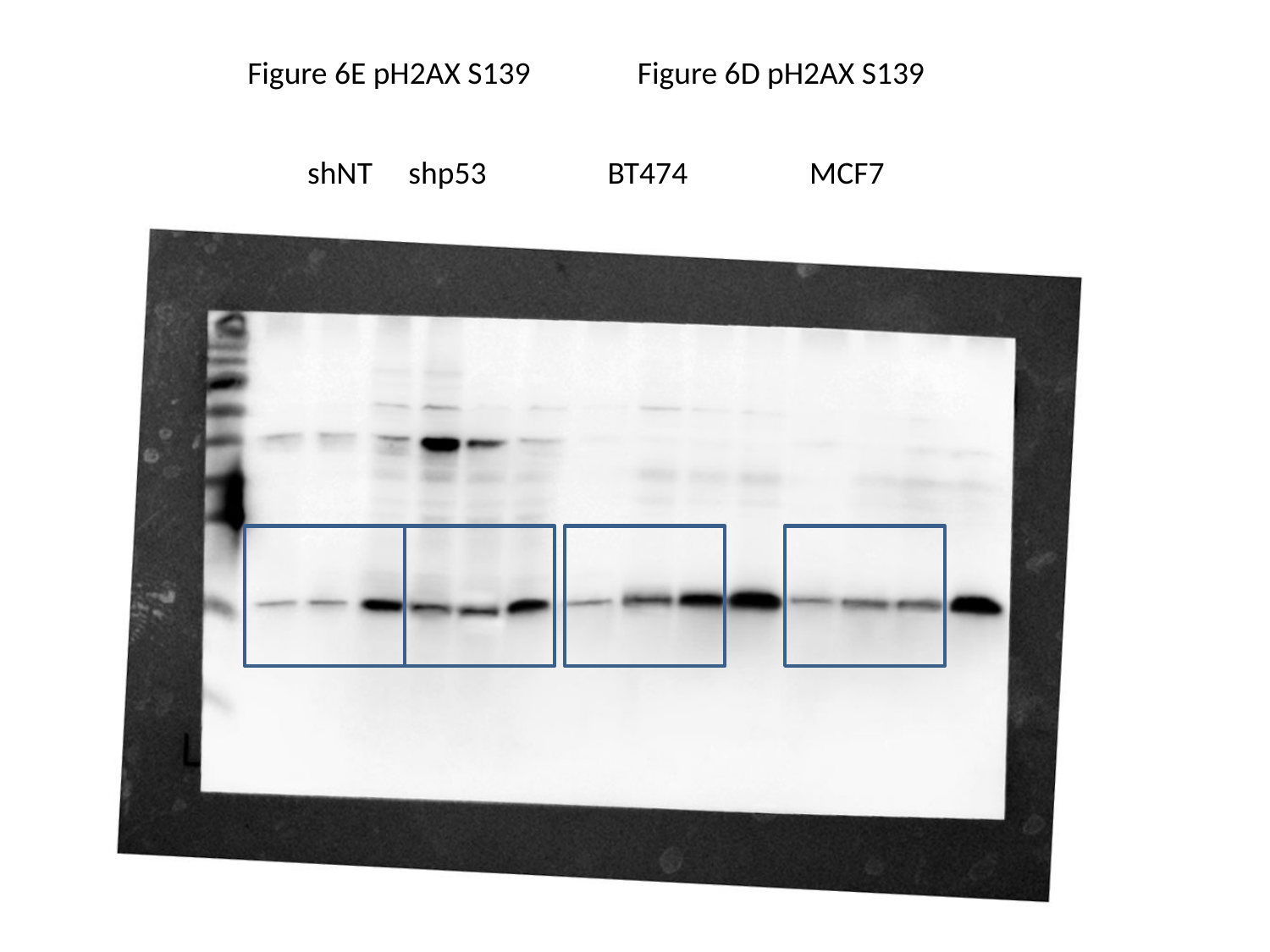

Figure 6E pH2AX S139
Figure 6D pH2AX S139
shNT shp53
BT474 MCF7

## Slide 14
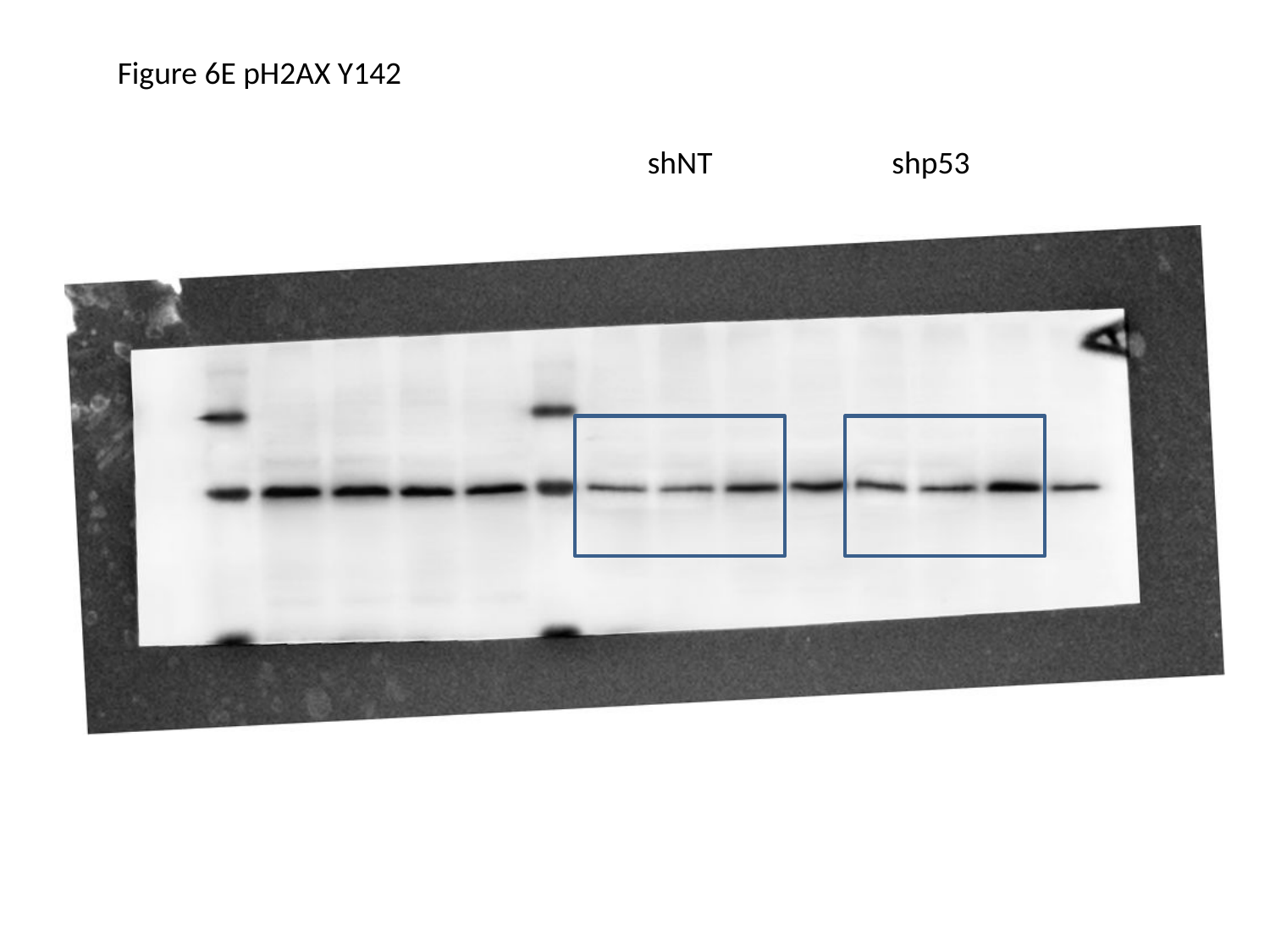

Figure 6E pH2AX Y142
shNT shp53

## Slide 15
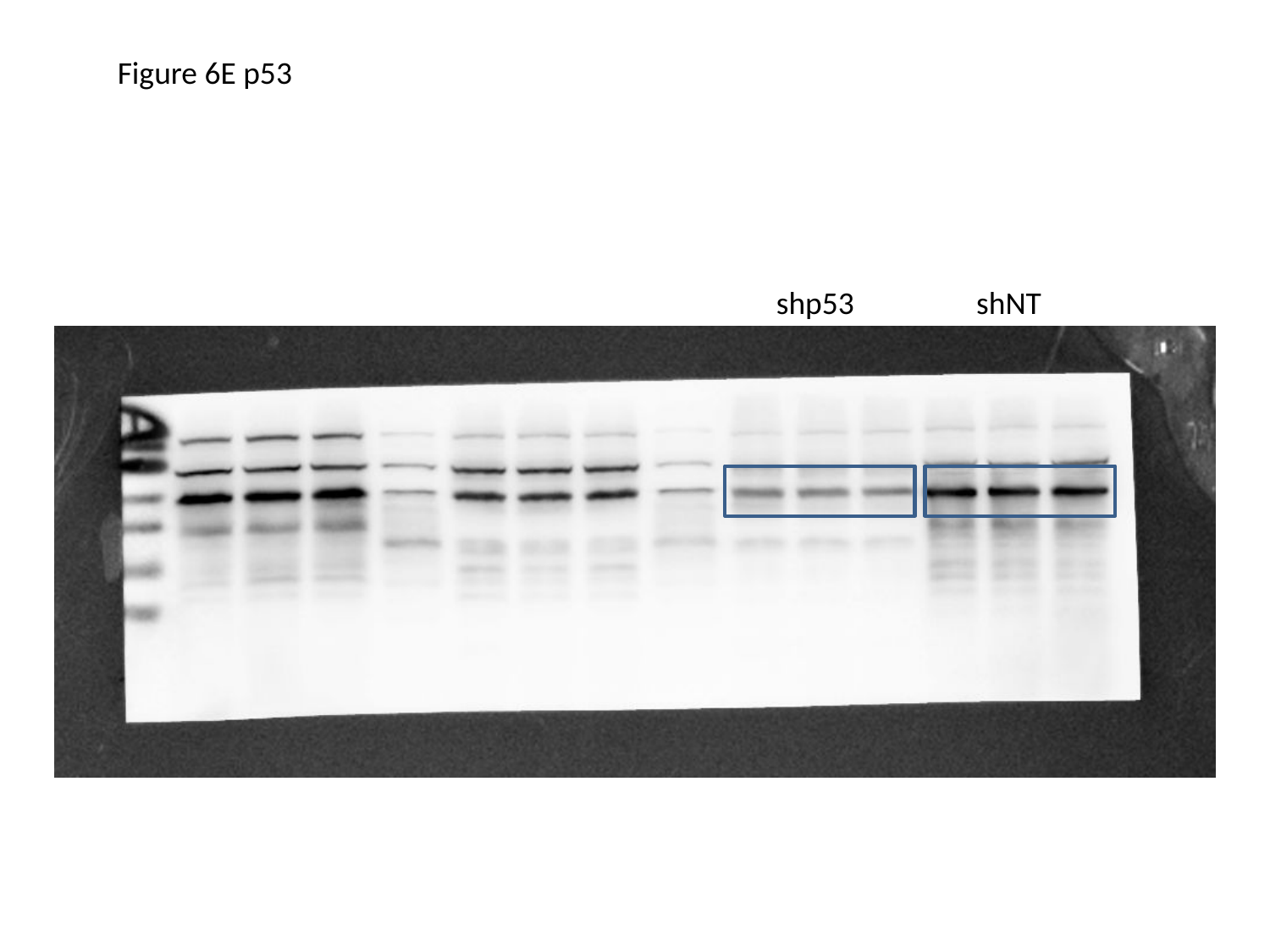

Figure 6E p53
 shp53 shNT

## Slide 16
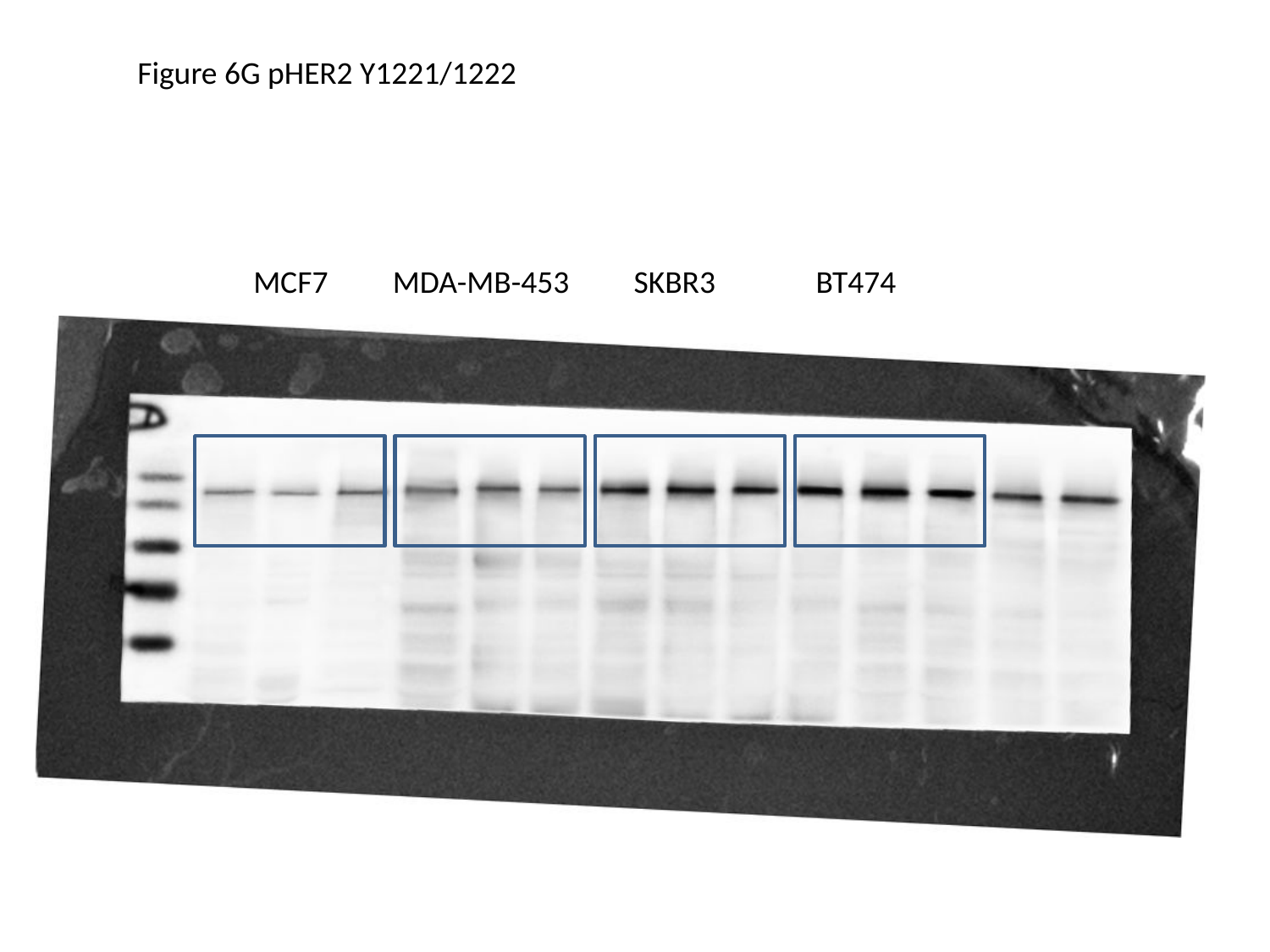

Figure 6G pHER2 Y1221/1222
 MCF7 MDA-MB-453 SKBR3 BT474

## Slide 17
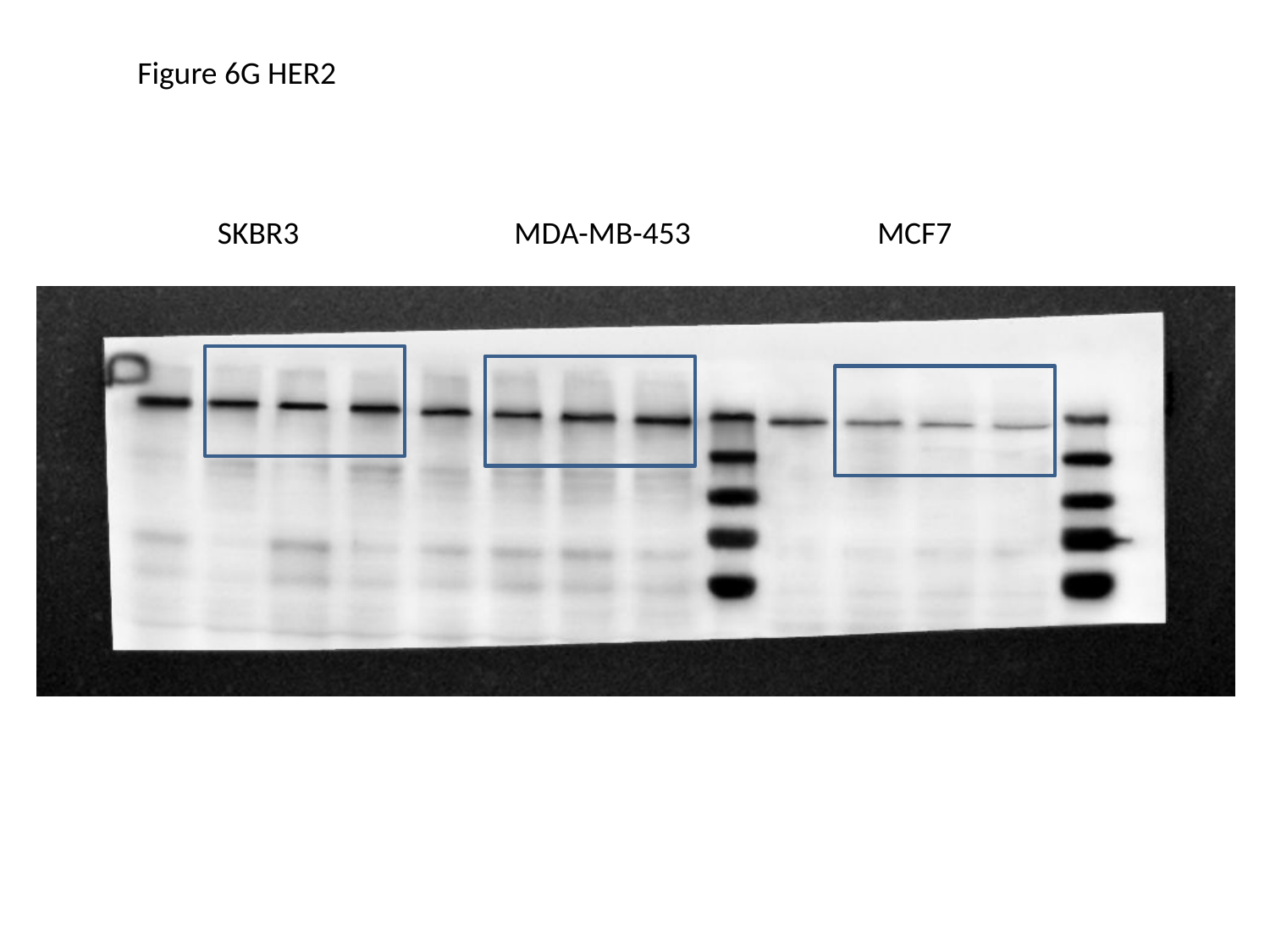

Figure 6G HER2
SKBR3 MDA-MB-453 MCF7

## Slide 18
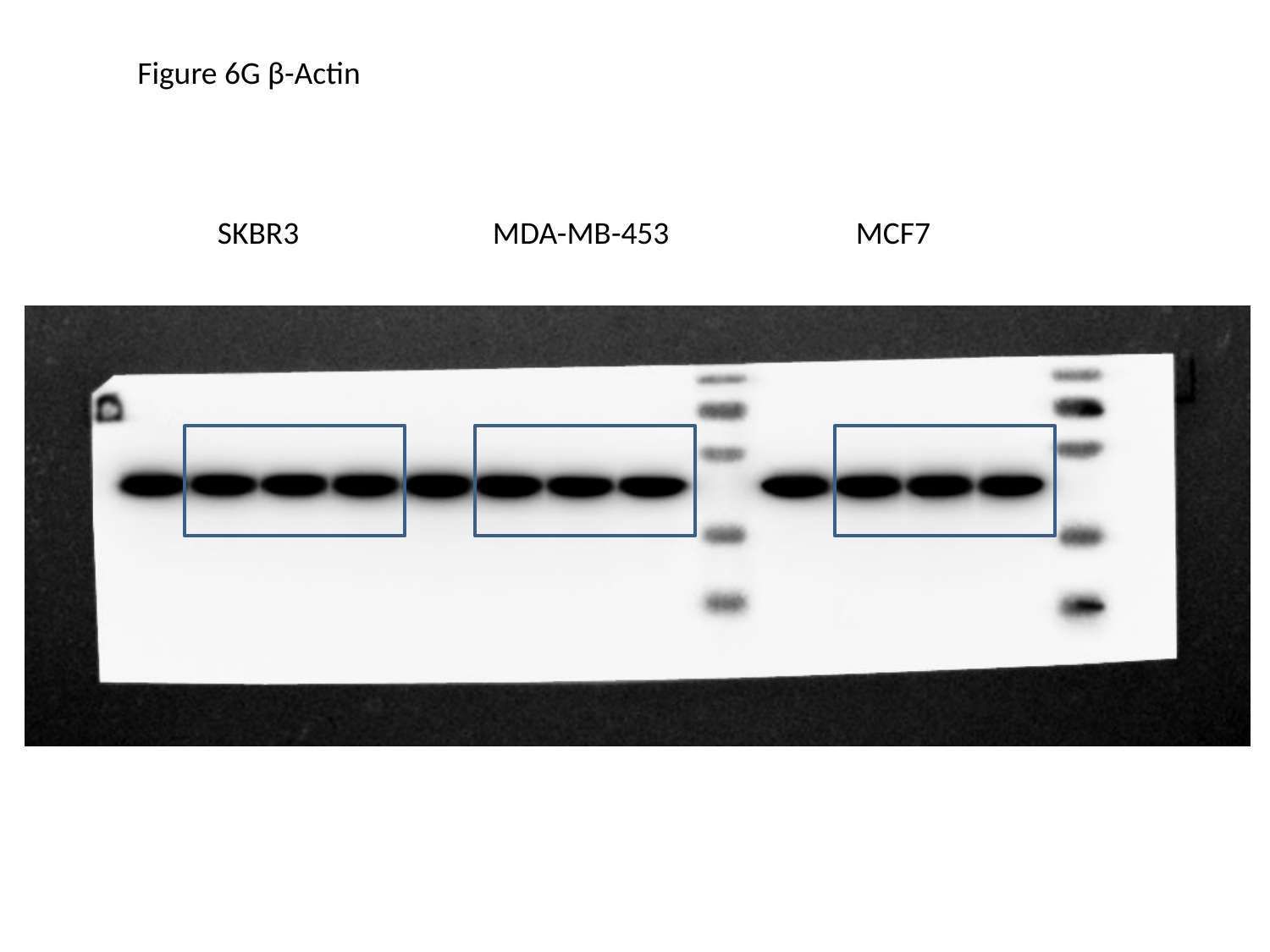

Figure 6G β-Actin
SKBR3 MDA-MB-453 MCF7

## Slide 19
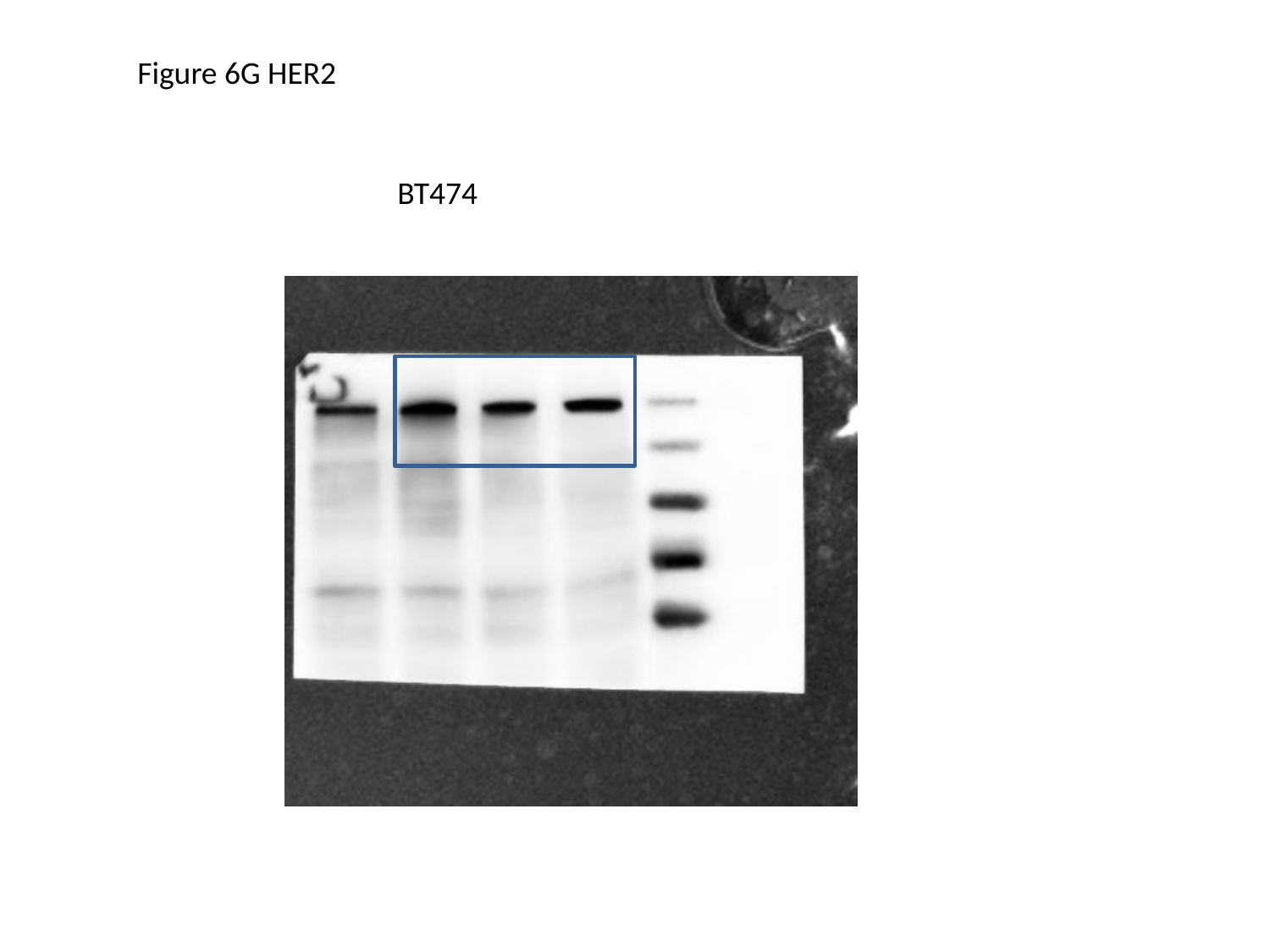

Figure 6G HER2
BT474

## Slide 20
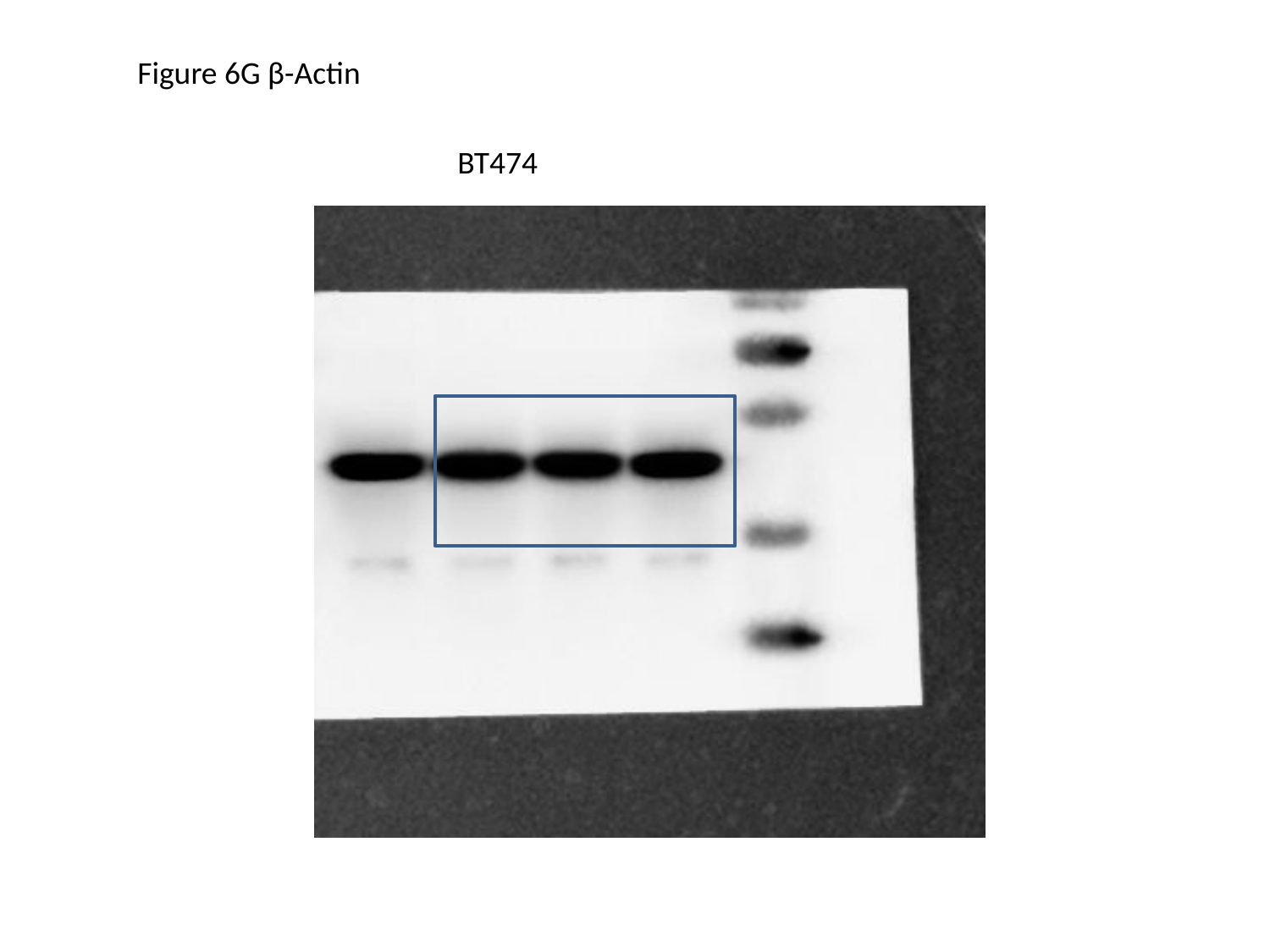

Figure 6G β-Actin
BT474
